# Src-mediated tyrosine phosphorylation of PRC1 and kinastrin/SKAP on the mitotic spindle

**DOI:** 10.1038/s41598-021-82189-1

**Published:** 2021-01-28

**Authors:** Mariko Morii, Sho Kubota, Chizu Hasegawa, Yumi Takeda, Shiori Kometani, Kyoko Enomoto, Takayuki Suzuki, Sayuri Yanase, Rika Sato, Aki Akatsu, Kensuke Hirata, Takuya Honda, Takahisa Kuga, Takeshi Tomonaga, Yuji Nakayama, Noritaka Yamaguchi, Naoto Yamaguchi

**Affiliations:** 1 Laboratory of Molecular Cell Biology, Graduate School of Pharmaceutical Sciences, Chiba University, Inohana 1-8-1, Chuo-ku, Chiba, 260-8675 Japan; 2 Laboratory of Transcriptional Regulation in Leukemogenesis, International Research Center for Medical Sciences (IRCMS), Kumamoto University, Kumamoto, 860-0811 Japan; 3 Laboratory of Proteome Research, National Institutes of Biomedical Innovation, Health and Nutrition, Ibaraki, Osaka 567-0085 Japan; 4 Department of Biochemistry and Molecular Biology, Kyoto Pharmaceutical University, Kyoto, 607-8414 Japan

**Keywords:** Biological techniques, Cell biology

## Abstract

Src-family tyrosine kinases (SFKs) play important roles in a number of signal transduction events during mitosis, such as spindle formation. A relationship has been reported between SFKs and the mitotic spindle; however, the underlying mechanisms remain unclear. We herein demonstrated that SFKs accumulated in the centrosome region at the onset of mitosis. Centrosomal Fyn increased in the G_2_ phase in a microtubule polymerization-dependent manner. A mass spectrometry analysis using mitotic spindle preparations was performed to identify tyrosine-phosphorylated substrates. Protein regulator of cytokinesis 1 (PRC1) and kinastrin/small kinetochore-associated protein (kinastrin/SKAP) were identified as SFK substrates. SFKs mainly phosphorylated PRC1 at Tyr-464 and kinastrin at Tyr-87. Although wild-type PRC1 is associated with microtubules, phosphomimetic PRC1 impaired the ability to bind microtubules. Phosphomimetic kinastrin at Tyr-87 also impaired binding with microtubules. Collectively, these results suggest that tyrosine phosphorylation of PRC1 and kinastrin plays a role in their delocalization from microtubules during mitosis.

## Introduction

Src-family kinases (SFKs) belong to the largest family of non-receptor-type tyrosine kinases and consist of at least eight highly homologous members, such as c-Src, Fyn, c-Yes, and Lyn^1,2^. SFKs play important roles in a number of cell signaling events, including proliferation, differentiation, and migration^3^. The expression profiles of SFKs in a single cell show specific and overlapping functions^1,2^. Although SFKs mainly localize to the cytoplasmic side of the plasma membrane^2^, some are present on intracellular organelle membranes, including late endosomes/lysosomes^4–8^, secretory granules^9^, and the Golgi apparatus^10–12^, besides the inside of the nucleus^13–17^. SFKs are associated with centrosomes^5,18^. c-Src has been detected in the centrosomes of NIH3T3 cells^18^, and Fyn localizes to the centrosome in interphase and to the spindle poles in metaphase in human T lymphocytes^5^.

Mitosis is a process by which a parent cell divides into two daughter cells, and is classified into the following phases: prophase, prometaphase, metaphase, anaphase, and telophase. The mitotic process is primarily controlled by posttranslational mechanisms, such as phosphorylation and proteolysis, and needs to be spatiotemporally regulated in a sophisticated manner. There is growing evidence to show that SFKs play significant roles in mitosis. We previously demonstrated that two SFKs, Lyn and c-Yes, their negative regulator tyrosine kinases, Csk and Chk, and some tyrosine-phosphorylated proteins were associated with mitotic chromosomes and spindle fibers, suggesting that SFK-mediated tyrosine phosphorylation is involved in chromosome dynamics^13^. c-Src, Fyn, c-Yes, and Lyn, which are co-expressed in HeLa cells, are all activated in the M phase^19,20^, and mitotic SFK activation leads to MEK/ERK activation for abscission in cytokinesis^21^. Furthermore, c-Src promotes proper spindle orientation^22^, while Fyn increases the rate of microtubule polymerization and accelerates mitotic progression^23^. v-Src, a viral oncogenic derivative of the proto-oncogene c-Src, has been shown to partly inhibit cell proliferation through the aberrant localization of Mklp1 and Aurora B, resulting in mitotic slippage, binucleation, and chromosome abnormalities^24–26^. The membrane attachment of SFKs via their lipid modifications during the period from prophase to anaphase plays a protective role against chromosome missegregation^27^. These findings support the possibility that SFKs have spatiotemporal roles in various mitotic processes. Although SFKs are reported to be associated with the centrosome^5,18^, limited information is currently available on the spatiotemporal localization of SFKs in the centrosome. Furthermore, tyrosine-phosphorylated substrates that are associated with mitotic spindles remain poorly understood.

We herein demonstrated that the SFK member Fyn accumulated in the centrosome region in cell-cycle- and microtubule-dependent manners. We performed an MS analysis to identify tyrosine-phosphorylated proteins associated with mitotic spindles and found a number of tyrosine phosphorylated proteins, including PRC1 and kinastrin. PRC1 at Tyr-464 and kinastrin at Tyr-87 were identified as two main tyrosine phosphorylation sites for SFKs. The results obtained also revealed that the tyrosine phosphorylation of PRC1 at Tyr-464 and kinastrin at Tyr-87 leads to their delocalization from microtubules during mitosis.

## Results

### Cell cycle-dependent centrosomal accumulation of SFKs

**Figure 1:**
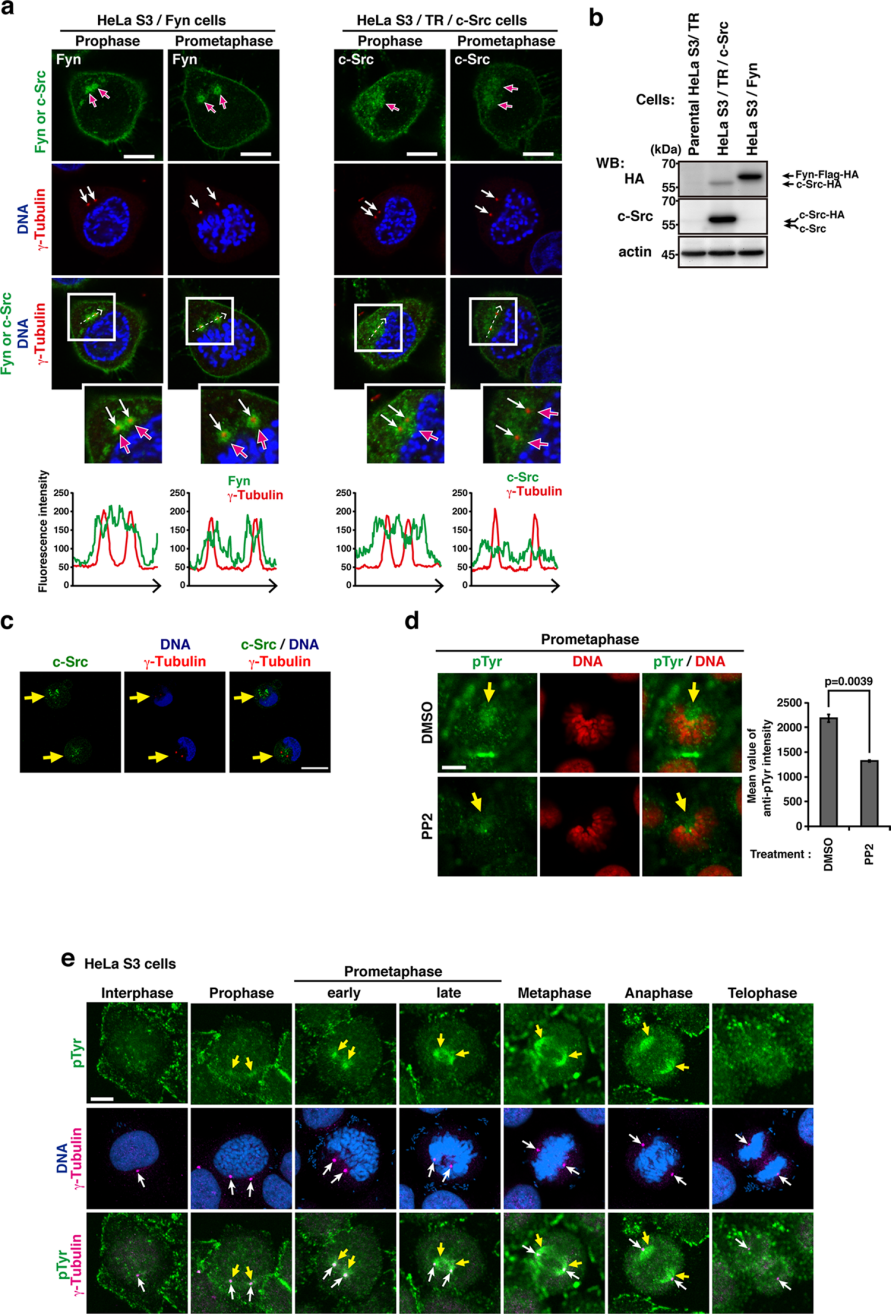
Accumulation of SFKs and tyrosine-phosphorylated proteins in the centrosome during G_2_ and M phases. **(a)** HeLa S3/Fyn or HeLa S3/TR/c-Src cells treated with or without Dox for inducible expression were triply stained for Fyn or c-Src (green), γ-tubulin (red), and DNA (blue). Cells entering the late G_2_ and M phases were assessed by chromosome condensation. Scale bars, 10 μm. **(b)** HeLa S3/TR, HeLa S3/TR/c-Src, or HeLa S3/Fyn cells were treated with or without Dox for inducible expression. A Western blot analysis was conducted using the indicated antibodies. **(c)** Centrosomal endogenous c-Src cells were triply stained for c-Src (green), γ-tubulin (red), and DNA (blue) in Caco-2 cells. Scale bar, 20 μm. **(d)** Parental HeLa S3 cells were treated with 20 µM PP2 or 0.1% dimethyl sulfoxide (DMSO, solvent control) for 6 h. Double staining for pTyr (green) and DNA (red) was performed and the fluorescence intensity of pTyr in the centrosome of each cell was quantitated from three independent experiments (n = 121 cells for DMSO, n = 118 cells for PP2 treatment). Bars represent the means ± S.D. of a representative experiment. *p* values were calculated by the Student’s *t*-test. Scale bar, 10 μm. **(e)** Parental HeLa S3 cells were triply stained for pTyr (green), γ-tubulin (red), and DNA (blue). Scale bar, 10 μm. **(a,c–e)** White, white with red, and yellow arrows indicate the centrosome position, centrosomal Fyn or c-Src accumulation, and endogenous tyrosine-phosphorylated proteins or endogenous c-Src in the centrosome, respectively.

Fyn localizes to the centrosome in interphase and to the spindle poles in metaphase in human T lymphocytes^5^. c-Src, a ubiquitously expressed SFK member, was also found in the centrosomal region in interphase^18^. We investigated whether the relationship between SFKs and the centrosome was influenced by cell cycle progression. To compare the localization of Fyn and c-Src, Fyn- or c-Src-expressing HeLa S3 cells (HeLa S3/Fyn or HeLa S3/TR/c-Src) arrested in the G_2_ phase were released into the M phase (Fig. 1a,b). Consistent with previous findings, centrosomal Fyn was observed in HeLa S3/Fyn (Fig. 1a). Immunostaining revealed that c-Src was more widely distributed than Fyn (Fig. 1a). Even though a number of anti-Fyn antibody reagents were commercially available to us, it was not possible to visualize endogenous centrosomal Fyn in HeLa cells despite their proper protein expression of Fyn^19,28^. To investigate the centrosomal localization of endogenous c-Src, we used Caco-2 cells, which strongly express c-Src, and found that c-Src also accumulated in the centrosome region (Fig. 1c). These results indicate that Fyn and c-Src associate with the centrosome.

We then performed co-immunostaining for Fyn and cyclin B1, a G_2_/M-phase marker^19,29^, and found that centrosomal Fyn increased as the expression of cyclin B1 was up-regulated (Suppl. Fig. S1a-c). Centrosomal Fyn was more prominent in synchronized cells, particularly with the thymidine → RO-3306 treatment, than in asynchronous cells (Suppl. Fig. S1d-i). Microtubules are nucleated by centrosomes and play indispensable roles in intracellular transport and organelle positioning^30^. To investigate the involvement of microtubules in centrosomal Fyn accumulation, cells were incubated with nocodazole or a cold treatment, which depolymerizes microtubules. Nocodazole or the cold treatment disrupted microtubules and the centrosomal accumulation of Fyn in prophase and prometaphase, while Aurora A remained in the centrosome (Suppl. Fig. S2a-c), which is consistent with previous findings^5^. HeLa S3/Fyn cells in the late G_2_ phase and early M phase were immunostained with the anti-Src[pY^416^] antibody, which shows SFKs in a kinase-active conformation. Kinase-active SFKs were clearly detected in the centrosome region in the indicated phases (Suppl. Fig. S3). These results suggest that kinase-active SFKs localize to the centrosome region in the G_2_ and early M phases.

### Tyrosine phosphorylation induced by SFKs in mitotic spindles

To examine the localization of tyrosine-phosphorylated proteins during the M phase, parental HeLa S3 cells were fixed/extracted in PTEMF buffer containing Na_3_VO_4_, a potent tyrosine phosphatase inhibitor^28,31^. Immunostaining with anti-phosphotyrosine (pTyr) revealed that the Na_3_VO_4_ treatment significantly increased the level of endogenous tyrosine phosphorylation in the centrosome region (Suppl. Fig. S4a). The levels of centrosomal tyrosine phosphorylation in prometaphase were significantly reduced by the treatment with the SFK inhibitor PP2 (Fig. 1d, Suppl. Fig. S4a, b), but not by that with the multi-targeted receptor tyrosine kinase inhibitor sunitinib^32^ or the Aurora A kinase inhibitor MLN8237^23,33^ (Suppl. Fig. S4a-d), suggesting that tyrosine phosphorylation in the centrosome region is dependent on SFK activity. Endogenous tyrosine-phosphorylated proteins were visualized not only in the centrosome region, but also on the mitotic spindle in prophase and prometaphase (Fig. 1e). Between metaphase and anaphase, tyrosine-phosphorylated proteins localized to the mitotic spindle and poles; however, Fyn was not detected in these areas. These results indicate that tyrosine-phosphorylated substrates of SFKs are distributed along mitotic spindle microtubules.

### Identification of tyrosine-phosphorylated proteins associated with mitotic spindles

**Figure 2:**
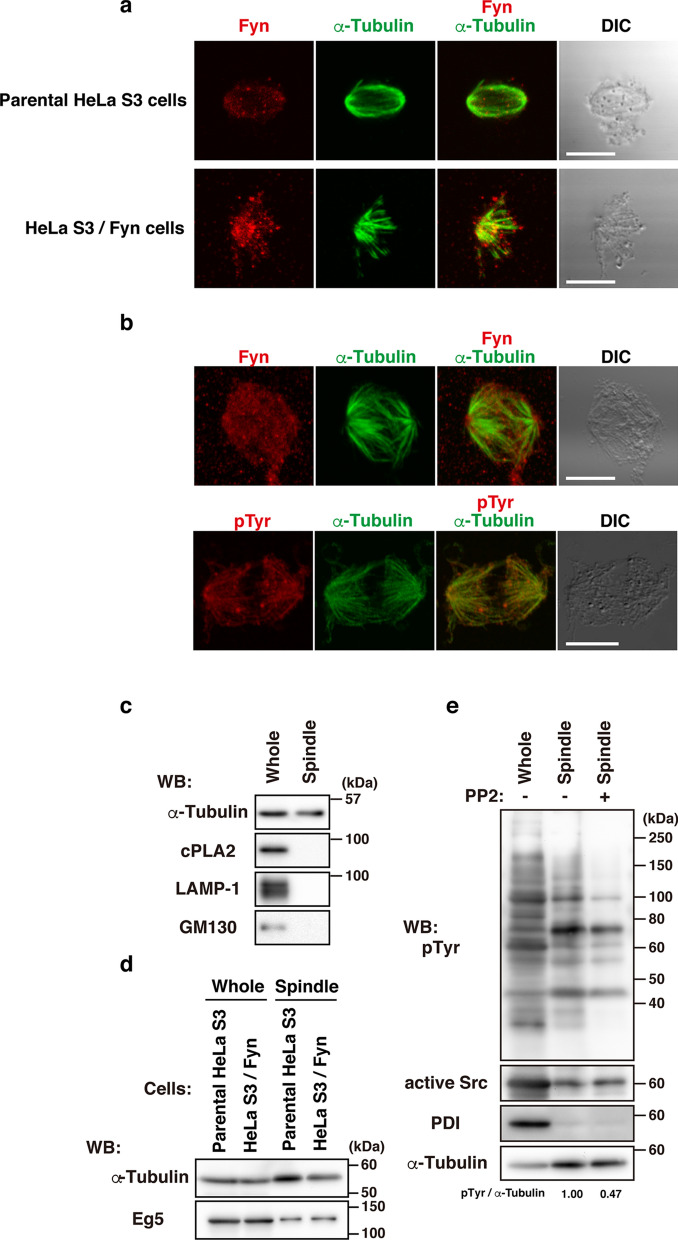
Tyrosine phosphorylation on mitotic spindles. Taxol-stabilized mitotic spindles were isolated from cells released from nocodazole-induced arrest (see “Methods”). **(a)** Mitotic spindles isolated from parental HeLa cells (upper panels) and HeLa S3/Fyn cells (lower panels) were doubly stained for endogenous Fyn (red) plus α-tubulin (green) (upper panels) and expressed Fyn (red) plus α-tubulin (green) (lower panels). Scale bar, 10 μm. **(b)** Mitotic spindles isolated from HeLa S3/Fyn cells were doubly stained for Fyn (red) plus α-tubulin (green) (upper panels) and pTyr (red) plus α-tubulin (green) (lower panels). Scale bars, 10 μm. **(c–e)** Mitotic spindles isolated from **(c)** HeLa S3/Fyn cells, **(d)** parental HeLa S3 and HeLa S3/Fyn cells, and **(e)** HeLa S3/Fyn cells treated with or without 10 µM PP2 were lysed and analyzed by Western blotting using the indicated antibodies. Cellular tyrosine phosphorylation levels are shown relative to that in each control sample following normalization with α-tubulin levels.

To more clearly visualize Fyn and its potential substrates in metaphase, we attempted to isolate mitotic spindles devoid of cellular membranous and cytosolic components (Fig. 2a–e; see “Methods”). The treatment with Na_3_VO_4_ ensured the highly sensitive detection of tyrosine phosphorylation on isolated mitotic spindles (Fig. 2a–e; see “Methods”). Since tyrosine phosphorylated proteins were more enriched in lysates upon treatment with the microtubule-stabilizing reagent taxol, we treated cells with taxol when they were released from nocodazole arrest. Endogenous and overexpressed Fyn were similarly visualized around the mitotic spindles in parental HeLa S3 cells and HeLa S3/Fyn cells, suggesting that a 0.1% Triton-X-100-resistant membranous structure harboring Fyn surrounds the mitotic spindle (Fig. 2a,b), whereas potential tyrosine-phosphorylated substrates of Fyn clearly colocalized to the mitotic spindle (Fig. 2b). Our mitotic spindle preparations did not contain cytosolic phospholipase A_2_ (cPLA_2_, a cytosolic marker protein), golgin subfamily A member 2 (GM130, a Golgi marker protein), protein disulfide-isomerase (PDI, an ER marker protein), or lysosome-associated membrane glycoprotein 1 (LAMP1, a lysosome marker protein), but did include α-tubulin and kinesin-like protein KIF11 (Eg5, a spindle-associated protein) (Fig. 2c–e). The PP2 treatment markedly decreased tyrosine phosphorylation levels in spindle lysates, indicating that mitotic spindle preparations contain SFK substrates (Fig. 2e).

To identify tyrosine-phosphorylated proteins associated with the mitotic spindle, tyrosine-phosphorylated proteins were purified from isolated spindle lysates by immunoprecipitation with the anti-pTyr antibody. Isolated proteins were digested with trypsin and tyrosine-phosphorylated peptides were detected by LC/MS/MS. This analysis identified 58 tyrosine-phosphorylated proteins, including spindle/microtubule-interacting proteins, such as protein regulator of cytokinesis 1 (PRC1) and kinastrin/small kinetochore-associated protein (kinastrin/SKAP), in addition to cyclin-dependent kinase 1 (Cdk1)^25,34^ and nuclear mitotic apparatus protein 1 (NuMA1)^35^ (Suppl. Table S1).

### Tyrosine phosphorylation of PRC1 at Tyr-464

**Figure 3:**
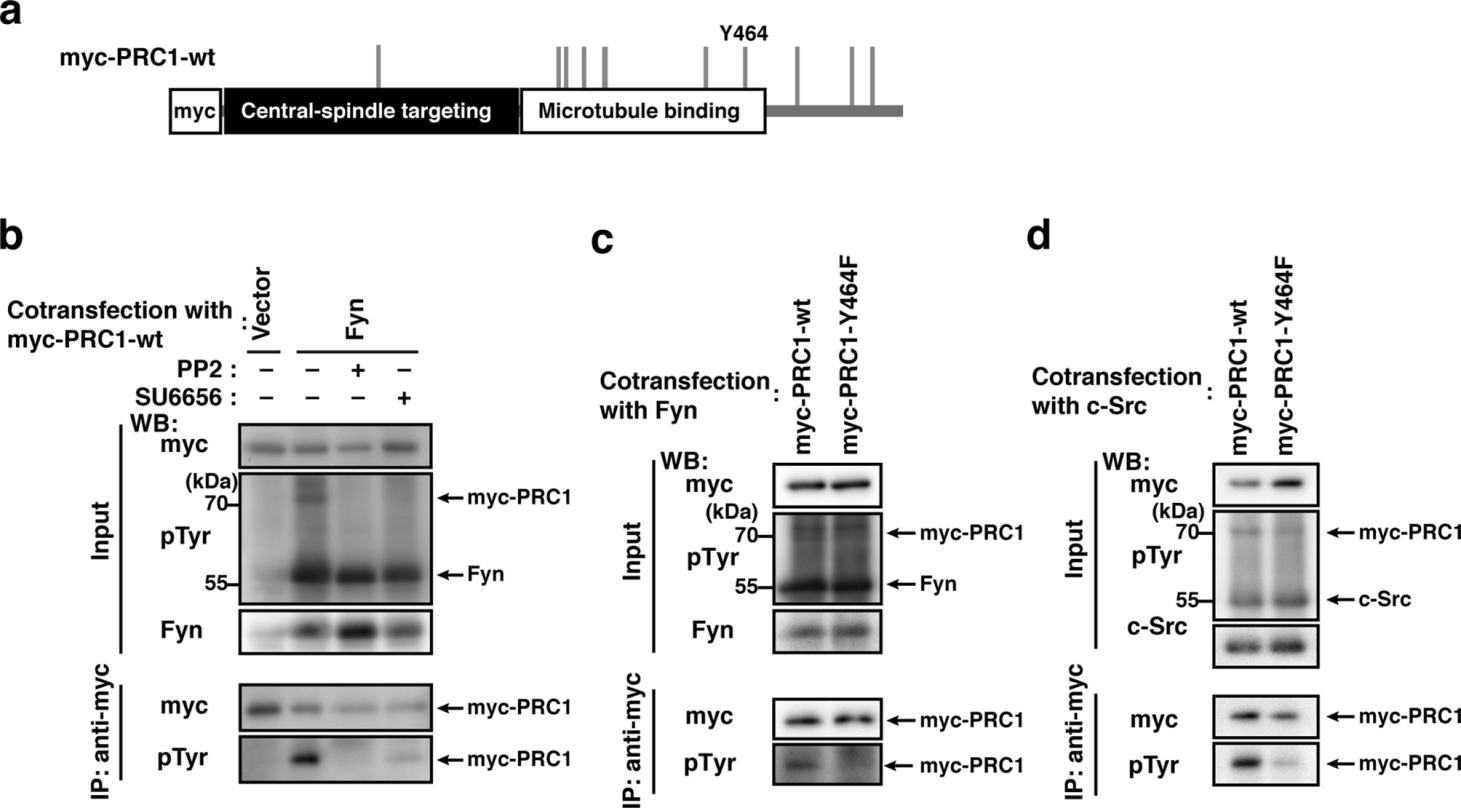
Tyrosine phosphorylation of PRC1 at Tyr-464. **(a)** A schematic representation of myc-PRC1-wt. Gray bars show tyrosine residue sites on PRC1. Y464 shows tyrosine residue sites mutated to phenylalanine or glutamate. **(b)** COS-1 cells co-transfected with the myc-PRC1-wt plus vector or Fyn were cultured for 24 h, and PP2, SU6656, or DMSO was added in the last 12 h. The immunoprecipitation of myc-PRC1-wt was performed with the anti-myc antibody. Western blotting was conducted using the indicated antibodies **(c,d)** COS-1 cells co-transfected with myc-PRC1-wt or myc-PRC1-Y464F plus Fyn **(c)** or c-Src **(d)** were cultured for 24 h. The immunoprecipitation of myc-PRC1-wt and the myc-PRC1-Y464F mutant was performed with the anti-myc antibody. Western blotting was conducted using the indicated antibodies.

PRC1, a microtubule-binding and -bundling protein, is a master regulator of normal cytokinesis^36^. The results of the phosphoproteomic analysis revealed a tyrosine-phosphorylated peptide of PRC1 possessing phospho-Tyr-464 (Fig. 3a, Suppl. Table S1). Intriguingly, Tyr-464 is located in close proximity to Thr-470 and Thr-481, both of which are known to maintain inactive PRC1 in early mitosis by Cdk phosphorylation^37–39^. To verify the tyrosine phosphorylation of PRC1, cells were co-transfected with PRC1-wt plus Fyn and cultured with or without the SFK inhibitor PP2 or SU6656. The tyrosine phosphorylation of PRC1-wt by Fyn was noted, and was suppressed by the application of PP2 or SU6656 (Fig. 3b). A PRC1 mutant with a phenylalanine substitution for Tyr 464 (Y464F) was generated (Fig. 3a) and its phosphorylation by Fyn and c-Src was examined. The phosphorylation of PRC1 by Fyn or c-Src was markedly reduced in the Y464F mutant (Fig. 3c,d). Therefore, Tyr-464 appears to be a major tyrosine phosphorylation site in PRC1.

### Role of the SFK-mediated tyrosine phosphorylation of PRC1 at Tyr-464

**Figure 4:**
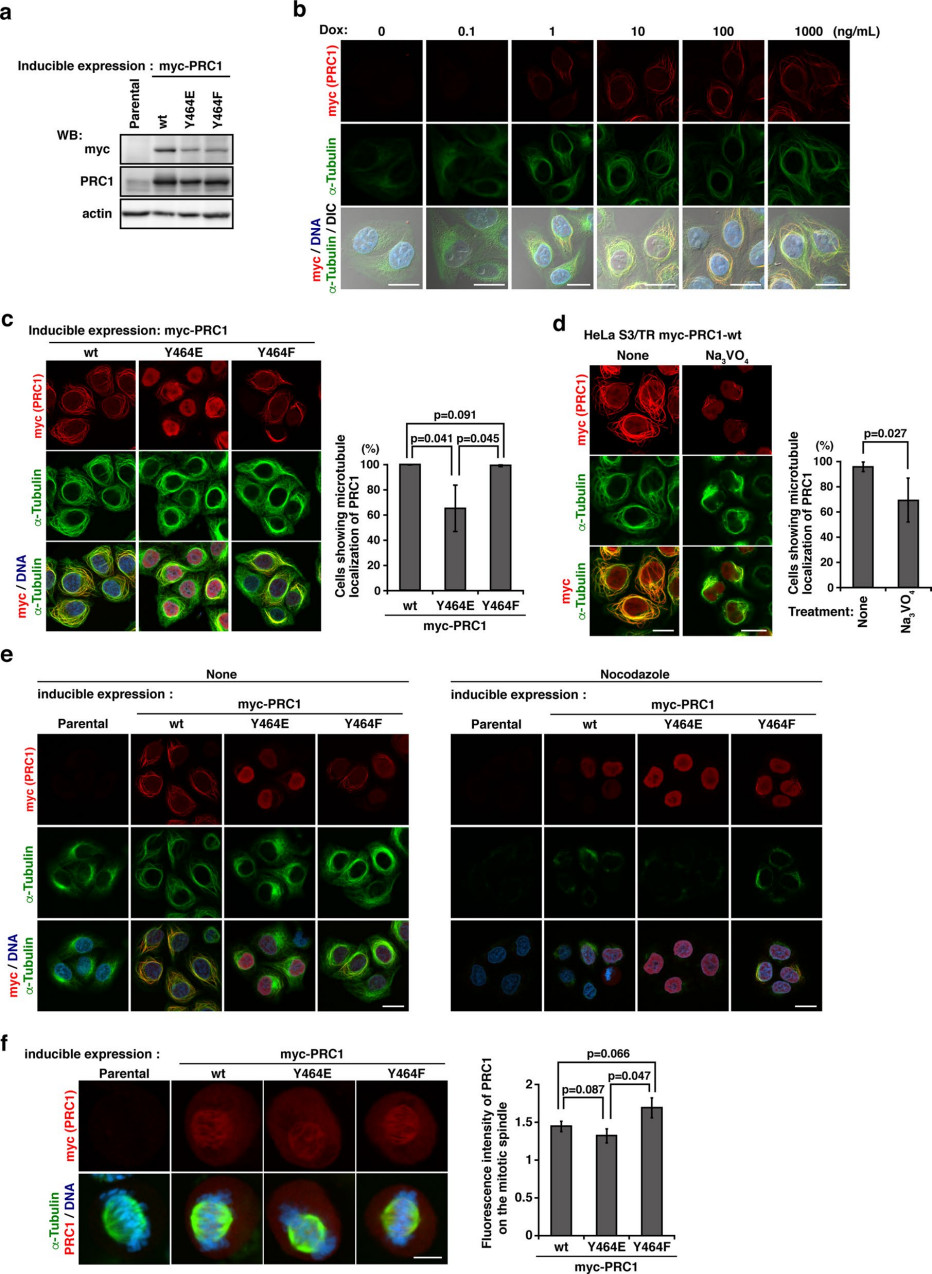
The effects of the tyrosine phosphorylation of PRC1 at Tyr-464 on its association with microtubules. **(a–e)** Parental HeLa S3/TR cells and HeLa S3/TR cells expressing myc-PRC1-wt, myc-PRC1-Y464E, or myc-PRC1-Y464F were treated with Dox for inducible expression. **(a)** A Western blotting (WB) analysis of whole cell lysates was conducted with the indicated antibodies. **(b–f)** Cells were stained for myc-PRC1, α-tubulin (green), and DNA. **(b)** Cells were treated with Dox at the indicated concentrations. Scale bars, 20 μm. **(c)** The percentage of cells showing the microtubule localization of myc-PRC1-wt, myc-PRC1-Y464E, or myc-PRC1-Y464F was assessed from three independent experiments (n = 402 cells for PRC1-wt, n = 211 cells for PRC1-Y464F, n = 308 cells for PRC1-Y464E). Scale bars, 20 μm. **(d)** Cells were treated with or without 1 mM Na_3_VO_4_. The percentage of cells showing the microtubule localization of myc-PRC1-wt was quantitated from four independent experiments (n = 489 cells for control, n = 487 cells for Na_3_VO_4_. treatment). Scale bars, 20 μm. **(e)** Parental HeLa S3/TR cells and HeLa S3/TR cells, which express myc-PRC1-wt, myc-PRC1-Y464E, or myc-PRC1-Y464F, were treated with Dox for inducible expression. Cells were treated with or without 1 µg/ml nocodazole for the last 30 min. Scale bars, 20 μm. **(f)** The fluorescence intensity of myc-PRC1-wt, myc-PRC1-Y464E, or myc-PRC1-Y464F on the mitotic spindle normalized by its fluorescence intensity in a whole cell (mean fluorescence intensity on the mitotic spindle/mean fluorescence intensity in a whole cell) was evaluated in three independent experiments (n = 8 cells for PRC1-wt, n = 7 cells for PRC1-Y464F, n = 9 cells for PRC1-Y464E). Scale bars, 10 μm.

PRC1, which exhibits microtubule-binding and -bundling activities, is essential for normal cytokinesis^38,39^. To clarify the involvement of the tyrosine phosphorylation of PRC1 at Tyr-464 in its relationship with microtubules, HeLa S3 cells that inducibly express PRC1-wt, its phosphodead Y464F mutant, and its phosphomimetic Y464E mutant were generated. The replacement of a tyrosine residue with a glutamate residue (YE) exerts phosphomimetic effects because the glutamate residue cannot be phosphorylated, but mimics permanent phosphorylation due to its negative charge. The treatment with Dox successfully induced the expression of PRC1-wt, PRC1-Y464F, and PRC1-Y464E (Fig. 4a). Like PRC1-wt, endogenous PRC1 associated with the mitotic spindle and mitotic spindle poles in early mitosis and with the spindle midzone in late mitosis (Suppl. Fig. S5a-c). During interphase, endogenous PRC1 mainly localized to the nucleus, and partially associated with microtubules, and PRC1-wt predominantly localized to the microtubules during interphase, which is consistent with previous findings^38,39^ (Fig. 4b). The localization of PRC1-Y464E to microtubules was significantly less than that of PRC1-wt and PRC1-Y464F (Fig. 4c). Similar to PRC1-Y464E, the Na_3_VO_4_ treatment significantly decreased the microtubule localization of PRC1-wt (Fig. 4d, Suppl. Fig. S5d). To examine the microtubule dependency of PRC1 cytoplasmic localization, cells were treated with nocodazole. The nocodazole treatment disrupted the cytoplasmic localization of PRC1-wt and PRC1-Y464F (Fig. 4e), indicating that PRC1-wt and PRC1-Y464F clearly associated with microtubules, as did PRC1-Y464E, but to a lesser extent. These results suggest that the tyrosine phosphorylation of PRC1 has a negative impact on the relationship between PRC1 and microtubules.

In metaphase, endogenous PRC1 was predominantly detected in the central part of metaphase spindles^40^. We examined the spindle localization of the PRC1 phosphomimetic and phosphodead forms at Tyr-464 in metaphase. The fluorescence intensity of PRC1-Y464E on the mitotic spindle was weaker than that of PRC1-Y464F (Fig. 4f), suggesting that the tyrosine phosphorylation of PRC1 at Tyr-464 impairs the relationship between PRC1 and metaphase spindles. Collectively, these results suggest that tyrosine phosphorylation of PRC1 at Tyr-464 plays a role in its delocalization from spindle microtubules.

### Tyrosine phosphorylation of kinastrin at Tyr-87

**Figure 5:**
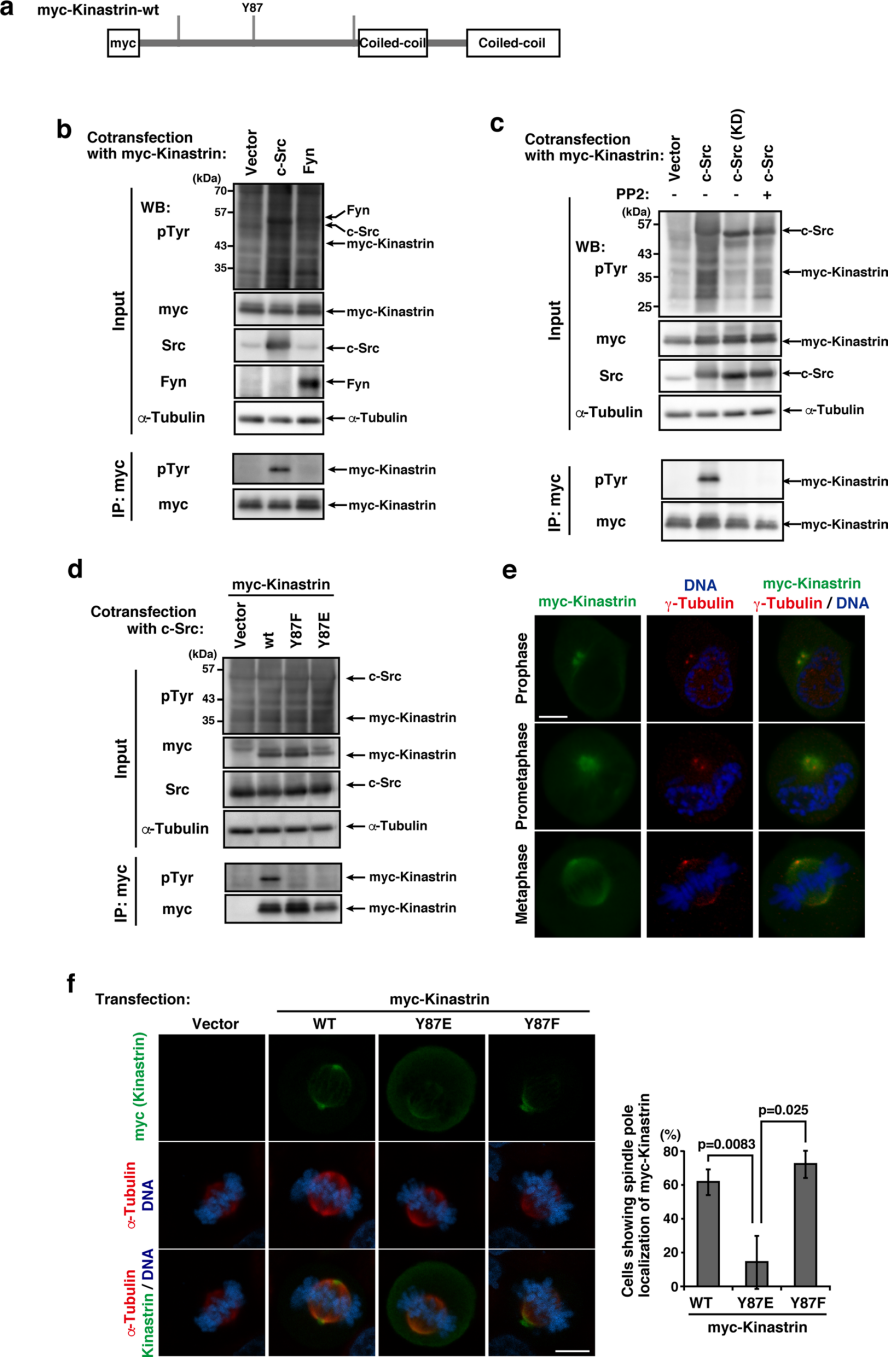
Tyrosine phosphorylation of kinastrin at Tyr-87. **(a)** A schematic representation of myc-kinastrin-wt. Gray bars show tyrosine residue sites on kinastrin. Y87 indicates tyrosine residue sites mutated to phenylalanine or glutamate. **(b)** COS-1 cells co-transfected with the myc-kinastrin-wt plus vector, Fyn, or c-Src were cultured for 24 h. **(c)** COS-1 cells co-transfected with the myc-kinastrin-wt plus vector or c-Src were cultured for 24 h, with the addition of PP2 or DMSO in the last 12 h. **(b,c)** The immunoprecipitation of myc-kinastrin-wt was performed with the anti-myc antibody. Western blotting was conducted using the indicated antibodies. **(d)** COS-1 cells co-transfected with myc-PRC1-wt or its mutants plus c-Src were cultured for 24 h. The immunoprecipitation of myc-kinastrin-wt and its mutants was performed using the anti-myc antibody. Western blotting was conducted using the indicated antibodies. **(e,f)** HeLa S3/TR cells transiently transfected with myc-kinastrin-wt or its mutants, were cultured with thymidine for 24 h, transferred for 4.5 h into fresh medium, and then treated with Dox for 7 h. Scale bars, 10 μm. **(e)** Cells were stained for myc-kinastrin (green), γ-tubulin (red), and DNA. **(f)** The percentage of cells showing the spindle localization of myc-kinastrin was assessed. Results (%) represent the means ± S.D. of three independent experiments (n = 164 cells for kinastrin-wt, n = 165 cells for kinastrin-Y464F, n = 158 cells for kinastrin-Y464E).

The results of the phosphoproteomic analysis revealed a tyrosine-phosphorylated peptide of kinastrin/SKAP containing phospho-Tyr-87 (Suppl. Table S1, Fig. 5a). Kinastrin is a kinetochore and mitotic spindle protein that plays many important roles in mitosis as the astrin/kinastrin complex^41^. To verify the tyrosine phosphorylation of kinastrin, cells were co-transfected with kinastrin (kinastrin-wt) plus Fyn or c-Src. The tyrosine phosphorylation of kinastrin-wt was clearly detected with the co-expression with c-Src, but not with Fyn (Fig. 5b). The treatment of cells with PP2 completely inhibited the tyrosine phosphorylation of kinastrin-wt (Fig. 5c). We also generated a kinastrin phosphomimetic mutant that has a glutamate substitution for Tyr-87 (Y87E) as well as a kinastrin phosphodead mutant with a phenylalanine substitution for Tyr-87 (Y87F) (Fig. 5a). Tyrosine phosphorylation of kinastrin by c-Src was undetected on kinastrin-Y87F and kinastrin-Y87E (Fig. 5d). These results suggest the possibility that Tyr-87 is a unique tyrosine phosphorylation site of kinastrin. To assess the influence of the tyrosine phosphorylation of kinastrin at Tyr-87 on their localization, HeLa S3/TR cells transfected with kinastrin-wt, its Y87F mutant, or its Y87E mutant were synchronized in the M phase. Kinastrin-wt expression was detected in spindle poles and spindle microtubules (Fig. 5e), which is consistent with previous findings^42^. The delocalization of kinastrin was more prominent in cells transfected with kinastrin-Y87E than in those with kinastrin-wt or kinastrin-Y87F (Fig. 5f). Therefore, the tyrosine phosphorylation of kinastrin at Tyr-87 appeared to impair the kinastrin association with spindle microtubules and spindle poles.

## Discussion

The present results revealed the accumulation of c-Src and Fyn in the centrosome region and the presence of tyrosine-phosphorylated substrates on mitotic spindles. A phosphoproteomic analysis with purified tyrosine-phosphorylated proteins on the mitotic spindle identified the mitotic spindle proteins PRC1 and kinastrin as novel SFK substrates. SFK-mediated tyrosine phosphorylation of PRC1 at Tyr-464 and kinastrin at Tyr-87 plays a role in delocalization of PRC1 and kinastrin from microtubules during mitosis.

Fyn localizes to the centrosome in interphase and to the mitotic spindle and poles in metaphase^5^. The present results showed that centrosomal Fyn significantly increased from the G_2_ phase. Fyn was detected in the mitotic poles in later prometaphase before the complete alignment of chromosomes. In contrast, Fyn was dispersed in metaphase to telophase (Suppl. Fig. S1). We also demonstrated that the centrosomal accumulation of Fyn occurred in a microtubule-dependent manner. Centrosomes are structurally complex organelles that recruit hundreds of proteins and perform many important functions. Based on the transition of Fyn during the M phase, we assumed that the centrosomal region at which Fyn localizes is an amorphous-like structure that surrounds mitotic centrioles. Two different concepts have recently been proposed for the mitotic pericentriolar material: a liquid-like structure or a more solid-like structure^43^. Based on the concept that a liquid-like structure allows many constituent molecules to diffuse and interact with one another efficiently through phase separation, the role of phase separation in the delocalization of Fyn from mitotic spindle poles in metaphase warrants further study.

Rab11-decorated recycling endosomes were recently identified as highly dynamic structures that organize around interphase centrosomes, mitotic spindles, and mitotic spindle poles. Rab11-endosomes may function as carriers of spindle pole proteins to mitotic spindle poles^44^. Evidence is emerging for the interaction between Fyn and Rab11 via UNC119a, which results in the completion of cytokinesis^45^. Since the centrosomal accumulation of Fyn was observed in a microtubule-dependent manner, Fyn may interact with Rab11-endosomes for the transition of Fyn during the M phase through centrosomal linker structures.

In addition to Fyn, c-Src, a ubiquitously expressed SFK member, was also detected in the centrosomal region in interphase^18^ (Fig. 1). Besides SFKs, Syk tyrosine kinase localizes to the centrosome during interphase and prophase, and centrosomal Syk levels significantly decrease during metaphase^46^. The phosphorylation levels of β-catenin at Tyr-142 by Syk^47^ and Fyn^48^ have been shown to increase in the centrosome during interphase, but abruptly decrease in metaphase^47^. These findings on centrosomal phospho-Tyr-142 β-catenin and centrosomal Fyn support our hypothesis that the centrosome serves as a platform for spatiotemporal tyrosine phosphorylation signaling.

The role of tyrosine phosphorylation in the mitotic spindle currently remains unclear. We showed that Fyn promoted microtubule polymerization and accelerated M-phase progression^23^, suggesting that SFKs regulate the function of mitotic spindle-associated proteins through tyrosine phosphorylation. Recent studies reported that tyrosine phosphorylation regulated Polo-like kinase 1 (PLK1) for the mitotic checkpoint and cell division^49^. SFKs have been shown to phosphorylate Aurora-A at Tyr-148, which increased Aurora-A kinase activity and centrosomal recruitment^50^. Furthermore, SFK-mediated tyrosine phosphorylation of the mitotic kinesin Eg5 regulated spindle morphology^51^. Fifty-eight candidate substrates of SFKs, including PRC1 and kinastrin, were identified in the present study. We also identified the microtubule-binding protein NuMA, which has been reported as an ABL1 (also known as c-Abl) substrate^35^. Similar to SFKs, PRC1 and kinastrin accumulated in the centrosomal region in prometaphase (Fig. 3–5), suggesting that SFKs phosphorylate potential substrates through spatiotemporal interactions in the centrosome region.

We previously identified KAP1/Trim28/Tif1β and Tif1γ/Trim33 as substrates of SFKs and c-Abl tyrosine kinases. The tyrosine phosphorylation of KAP1 and Tif1γ weakened their relationships with binding partners and altered their localization^16,31^. The present results revealed that SFKs phosphorylate PRC1 at Tyr-464, which has a negative impact on its ability to associate with microtubules (Figs. 3, 4). Since the overexpression of PRC1 was detected in bundling microtubules^38,39^, resulting in aberrant spindle formation (Fig. 4e), the regulation of PRC1 activation in a spatiotemporal manner is important. The tyrosine-phosphorylation site of PRC1 is in close proximity to the Cdk phospho-threonines, Thr-470 and Thr-481, at the junction between the microtubule-binding domain and C-terminal region^37–39^. The Cdk-mediated phosphorylation of PRC1 in early mitosis maintains PRC1 in an inactive state. The dephosphorylation of PRC1 at Thr-470 and Thr-481 plays an essential role in regulating the bundling of interdigitating microtubules in order to establish the midzone, which is necessary for cytokinesis. Therefore, we assume that PRC1 phosphorylation at Tyr-464 works in cooperation with Cdk phosphorylation at Thr-470 and Thr-481 to maintain PRC1 in an inactive state in early mitosis.

We also identified kinastrin as an SFK substrate (Fig. 5). We detected the tyrosine phosphorylation of kinastrin by c-Src in interphase using a co-transfection experiment. Among three tyrosine residues, the substitution of Tyr-87 to phenylalanine or glutamic acid markedly decreased tyrosine phosphorylation. Kinastrin is expressed as two distinct isoforms in mammals: shorter and longer isoforms^42^. The present results obtained using the longer isoform of kinastrin suggested that the tyrosine phosphorylation of kinastrin decreases its localization to the mitotic spindle and poles. Since Tyr-87 is conserved in both isoforms, the role of the SFK-mediated tyrosine phosphorylation of the shorter form of kinastrin in the kinetochore association warrants further study.

The results of the phosphoproteomic analysis revealed that the tyrosine-phosphorylated proteins detected in the isolated mitotic spindle preparation contained various RNA-binding proteins and RNA-modifying enzymes at a ratio of one third or more (Suppl. Table S1). If these RNA-associated proteins are functionally regulated by tyrosine phosphorylation, as yet unidentified non-coding and/or coding RNAs may be closely involved in spindle formation and mitosis. Accordingly, tyrosine phosphorylation generated from the centrosome may play a number of roles in complex mitotic progression. In addition, the actin-binding proteins and intermediate filaments/their related proteins identified in the present study may be involved in spindle formation and mitosis through tyrosine phosphorylation (Suppl. Table S1).

In conclusion, we herein demonstrated that Fyn, a member of the SFK family, accumulated in the centrosome region in cell cycle- and microtubule-dependent manners. The present results identified several phosphorylated proteins that are associated with the mitotic spindle, and revealed their potential phosphorylation sites. Further studies will provide a more detailed understanding of the roles of SFK-mediated tyrosine phosphorylation during the M phase.

## Methods

### Plasmids

cDNAs encoding human Fyn^52^ (a gift from T. Yamamoto) and cDNA encoding human c-Src^53^ (a gift from D. J. Fujita) were ligated into the pcDNA4/TO vector^6,12,13,23^. PRC1 and kinastrin were cloned by PCR from human cDNAs, and the PCR product was ligated into the pcDNA4/myc vector^17^. The mutant cDNAs of PRC1 or kinastrin, which have phenylalanine or glutamic acid substitutions for tyrosine residues, were generated by site-directed mutagenesis using PCR. The primers used for PCR are shown in Suppl. Table S2.

### Cell lines and transfection

HeLa S3 cells (JCRB Cell Bank, Osaka) were grown in Iscove’s modified Dulbecco’s medium supplemented with 5% bovine serum. Plasmids were transfected using polyethylenimine (Polyscience, Inc.), as described previously^54^. HeLa S3 cells overexpressing Fyn tagged with FLAG-HA (FH) epitopes (HeLa S3/Fyn) and HeLa S3/TR cells inducibly expressing c-Src tagged with HA (HeLa S3/TR/c-Src) were generated previously^22,23,28^. HeLa S3/TR cells inducibly expressing Fyn (HeLa S3/TR/Fyn) were generated using HeLa S3/TR cells, which constitutively express the tetracycline repressor (TR)^24^, and pcDNA4/TO/neo/Fyn, as previously described^22^. Strictly controlled inducible expression is advantageous because protein expression levels may be adjusted to an appropriate amount^24,26^. SFK-mediated tyrosine phosphorylation was assessed using 20 µM PP2 (Sigma-Aldrich), 10 µM SU6656, 0.6 µM MLN8237 (Selleck Chemicals), and 10 µM sunitinib (Sigma-Aldrich).

### Cell synchronization

HeLa S3 cells can be precisely synchronized at specific cell cycle phases^20–22^. (i) (G_1_/S phase) Cells were cultured for 20–24 h with 4 mM thymidine (thymidine block)^55^. Alternatively, cells were cultured with 4 mM thymidine for 20 h, transferred into fresh medium for 9 h, and then cultured with 4 mM thymidine (double thymidine block) for 15 h^26^. (ii) (Late G_2_ phase) Cells arrested in the G_1_/S phase by the thymidine block were released for 9 h, and then treated with 9 μM of the Cdk1 inhibitor RO-3306 for 4–9 h^22^. Alternatively, cells were cultured with 9 µM RO-3306 for 20 h^22,23^. (iii) (M phase) Cells arrested in the G_1_/S phase or late G_2_ phase were cultured for the following times. Regarding the short-term Dox-induced expression of Fyn in HeLa S3/TR/Fyn cells, G_1_/S-arrested cells subjected to the double thymidine block were treated with Dox during the last 13-h incubation of the 2^nd^ thymidine treatment, and cells then were transferred into fresh medium containing Dox for 11 h. Concerning short-term PRC1 expression, HeLa S3/TR/PRC1-wt, -Y464E, and -Y464F cells were synchronized with the double thymidine block and then transferred into fresh medium containing Dox for the last 13 h. To achieve the short-term Dox-induced expression of myc-kinastrin, HeLa S3/TR cells transiently transfected with myc-kinastrin-wt or its mutants were cultured with thymidine for 24 h, transferred for 4.5 h into fresh medium, and then treated with Dox for 7 h.

### Antibodies

The following antibodies were used: Fyn (#25; BD Biosciences, and Fyn3; Santa Cruz Biotechnology), c-Src (#327; Calbiochem), Src[pY^416^] (phospho-Src family, Cell Signaling Technology), γ-tubulin (GeneTex, and clone GTU-88; Sigma-Aldrich or Abcam), phosphotyrosine (pTyr) (4G10; Upstate Biotechnology, Inc.; a gift from T. Tamura and T. Yoshimoto), IAK1 (Aurora A) (#4; BD Biosciences), phospho-Aurora A(Thr288)/Aurora B(Thr232)/Aurora C(Thr198) (pAurora A/B/C) (D13A11; Cell Signaling Technology), HA (F7 and Y-11; Santa Cruz Biotechnology), myc (9E10; Santa Cruz Biotechnology and A-14-G; Santa Cruz Biotechnology), α-tubulin (MCA78G; Serotec), cyclin B1 (H-433; Santa Cruz Biotechnology), PRC1 (H-70; Santa Cruz Biotechnology), GM130 (#35; BD Transduction Laboratories), cPLA_2_ (4-4B-3C; Santa Cruz Biotechnology), LAMP-1 (H4A3; Santa Cruz Biotechnology), and PDI (P7496; Sigma). Horseradish peroxidase-linked IgG F(ab’)_2_ and Alexa Fluor 488-, 546-, or 647-labeled IgG secondary antibodies were obtained from Amersham Biosciences and Invitrogen.

### Immunostaining

Confocal and differential-interference contrast (DIC) images were acquired using FV500 (Olympus) and LSM510 (Zeiss) laser scanning microscopes, as previously described^22,56^. (i) Cells were fixed at room temperature for 20 min in phosphate-buffered saline (PBS) containing 4% paraformaldehyde or PTEMF buffer (20 mM PIPES, pH 6.9, 0.2% Triton X-100, 10 mM EGTA, 1 mM MgCl_2_, and 4% paraformaldehyde)^17,22,23^, except in the following cases. (ii) To simultaneously detect Fyn and γ-tubulin, cells were fixed/extracted in PBS containing 20% methanol and 4% paraformaldehyde at room temperature for 20 min, and then treated with 100% methanol at − 30 °C for 5 min^55^. Alternatively, cells were fixed/extracted in PBS containing 0.02% saponin and 4% paraformaldehyde for 20 min. (iii) To detect tyrosine phosphorylation, cells were fixed/extracted in PTEMF buffer supplemented with 10 mM Na_3_VO_4_ and 10 mM unbuffered HEPES, and further extracted/blocked in PBS supplemented with 10 mM Na_3_VO_4_, 10 mM unbuffered HEPES, 0.1% saponin, and 3% bovine serum albumin (BSA). Na_3_VO_4_ was freshly prepared, as recently described^28,31^. (iv) Cells subjected to α-tubulin staining were fixed in PBS containing 4% paraformaldehyde or PTEMF buffer at 37 °C. Fixed cells prepared in (i)–(iv) were further extracted/blocked in PBS containing 0.1% saponin and 3% BSA and then immunostained with primary and secondary antibodies. Following the treatment with 200 µg/ml RNase A, DNA was stained with 20 nM TO-PRO-3 or 10–50 µg/ml propidium iodide for 30 min. Individual images were assembled using GIMP 2.6.2 and Illustrator 16.0 (Adobe). In addition to γ-tubulin, we used Aurora-A as a marker of centrosomes because it localizes to centrosomes from the S phase and then degrades in the early G_1_ phase^57^.

In Suppl. Fig. S1a and S1h, cells entering the late G_2_ and M phases were assessed by chromosome condensation^58^. In Suppl. Fig. S1c and S1e, the percentage of cells showing the centrosomal accumulation of Fyn was evaluated. In Fig. 5f, the percentage of cells showing the spindle localization of myc-kinastrin was quantitated. To quantify pTyr (Fig. 1d, Suppl. Fig. S4) and myc-PRC1 (Fig. 4f), the fluorescence intensities of immunostaining were measured using ImageJ software (National Institutes of Health). Since two or three independent experiments gave similar results, a representative experiment was shown. Bars represent the means ± S.D. of a representative experiment from three independent experiments. *p* values were calculated by the Student’s *t*-test.

### Microtubule depolymerization

In Fig. 4e, cells were treated with 1 µg/ml nocodazole for 30 min. In Suppl. Fig. S2, late G_2_ phase-arrested cells by the RO-3306 treatment for 20 h were transferred for 15 min into fresh medium containing 100 ng/ml nocodazole or were incubated on ice for the last 2 h (the cold treatment)^23^. Microtubule depolymerization was confirmed by α-tubulin immunostaining.

### Isolation of mitotic spindles

The procedure for spindle isolation was modified from that previously described^13,59^. HeLa S3/Fyn cells were cultured for 18 h with 100 ng/ml nocodazole. After mitotic shake-off, the cells collected were washed to remove nocodazole and cultured for 1 h with 10 µg/ml taxol to stabilize mitotic spindles together with 10 mM Na_3_VO_4_ to inhibit protein-tyrosine phosphatases. Cell pellets were lysed in isolation buffer [2 mM PIPES, pH 6.9, 5 µg/ml taxol, 10 mM Na_3_VO_4_, 10 mM unbuffered HEPES, 10 µg/ml latrunculin B, 0.1% Triton X-100, 70 U/ml DNase I, 10 µg/ml RNase A, 10 mM MgCl_2_, and protease inhibitors (1 mM PMSF, 50 µg/ml leupeptin, 25 µg/ml pepstatin A, and 50 µg/ml aprotinin)]. To remove contaminant chromosomes, membranous structures, and cytoplasmic components, isolated mitotic spindles were repeatedly washed with isolation buffer and then solubilized in RIPA buffer (50 mM HEPES, pH 7.4, 0.05% SDS, 1% Triton X-100, 0.2% deoxycholate, 10 mM Na_3_VO_4_, 10 mM unbuffered HEPES, and protease inhibitors).

### Identification of tyrosine-phosphorylated proteins associated with mitotic spindles

Isolated mitotic spindles prepared as described above were suspended in SDS lysis buffer (50 mM Tris–HCl, pH 7.5, 0.1% SDS, 10 mM Na_3_VO_4_, 10 mM unbuffered HEPES, 1 mM EGTA, 1 mM PMSF, 4 µg/ml aprotinin, 4 µg/ml leupeptin, and 1.6 µg/ml pepstatin A), sonicated, and heated at 95 °C for 5 min. After the addition of Triton X-100 at a final concentration of 0.5% to neutralize SDS, tyrosine-phosphorylated proteins were purified from the resulting lysate using the anti-pTyr antibody, as previously described^28^. Trypsinized peptides derived from tyrosine-phosphorylated proteins were identified by LC/MS/MS (see Suppl. Table S1), as previously described^16,17,28,31^.

### Immunoprecipitation and Western blot analysis

Cells were lysed in SDS lysis buffer and centrifuged to remove insoluble cellular components. The resulting supernatant was mixed with the appropriate antibody together with Protein G-Sepharose beads (GE Healthcare). After being incubated, beads were washed three times and then suspended in SDS-sample buffer. A Western blot analysis was performed with ECL (Millipore) as previously described^16,28^. SDS/PAGE was performed using whole-cell lysates in SDS-sample buffer and separated proteins were electrotransferred onto PVDF membranes. Appropriate antibodies were applied to detect protein bands, which were analyzed using ChemiDoc XRSPlus (Bio-Rad Laboratories).

## Supplementary Information

Supplementary Information.
