# Supplementary material for: Src-mediated tyrosine phosphorylation of PRC1 and kinastrin/SKAP on the mitotic spindle
Source: Sci Rep. 2021 Jan 28;11:2616. doi: 10.1038/s41598-021-82189-1 (PMC7844303; doi:10.1038/s41598-021-82189-1)
Supplement: Supplementary file 1 — Supplementary Information. [file 41598_2021_82189_MOESM1_ESM.pdf]

Supplementary information

Src-mediated tyrosine phosphorylation of PRC1 and kinastrin/SKAP on the mitotic spindle

Mariko Morii[1,2], Sho Kubota[1,2], Chizu Hasegawa[1], Yumi Takeda[1], Shiori Kometani[1], Kyoko Enomoto[1], Takayuki Suzuki[1], Sayuri Yanase[1], Rika Sato[1], Aki Akatsu[1], Kensuke Hirata[1], Takuya Honda[1], Takahisa Kuga[3], Takeshi Tomonaga[3], Yuji Nakayama[4], Noritaka Yamaguchi[1], and Naoto Yamaguchi[1,*]

[1]Laboratory of Molecular Cell Biology, Graduate School of Pharmaceutical Sciences, Chiba University, Chiba 260-8675, Japan
[2]Laboratory of Transcriptional Regulation in Leukemogenesis, International Research Center for Medical Sciences (IRCMS), Kumamoto University, Kumamoto 860-0811, Japan
[3]Laboratory of Proteome Research, National Institutes of Biomedical Innovation, Health and Nutrition, Ibaraki, Osaka 567-0085, Japan
[4]Department of of Biochemistry and Molecular Biology, Kyoto Pharmaceutical University, Kyoto 607-8414, Japan

*Corresponding author: Naoto Yamaguchi, Ph.D.
Laboratory of Molecular Cell Biology
Graduate School of Pharmaceutical Sciences
Chiba University
Inohana 1-8-1, Chuo-ku, Chiba 260-8675, Japan
E-mail: nyama@faculty.chiba-u.jp

Suppl. Fig. S1. Accumulation of Fyn in the centrosome region during G_2 and M phases.
Suppl. Fig. S2. Microtubule-dependent centrosomal accumulation of Fyn.
Suppl. Fig. S3. Centrosomal accumulation of kinase-active Src kinases.
Suppl. Fig. S4. Effect of kinase inhibitors on tyrosine phosphorylation in the centrosome region.
Suppl. Fig. S5. Effect of tyrosine phosphorylation on the microtubule localization of endogenous PRC1.
Suppl. Fig. S6. Full-length blots.
Suppl. Fig. S7. Full-length blots.
Suppl. Fig. S8. Full-length blots.
Suppl. Fig. S9. Full-length blots.
Suppl. Table 1. Identification of tyrosine-phosphorylated proteins in the isolated mitotic spindle preparation.
Suppl. Table 2. Primer list for PRC1 and kinastrin.

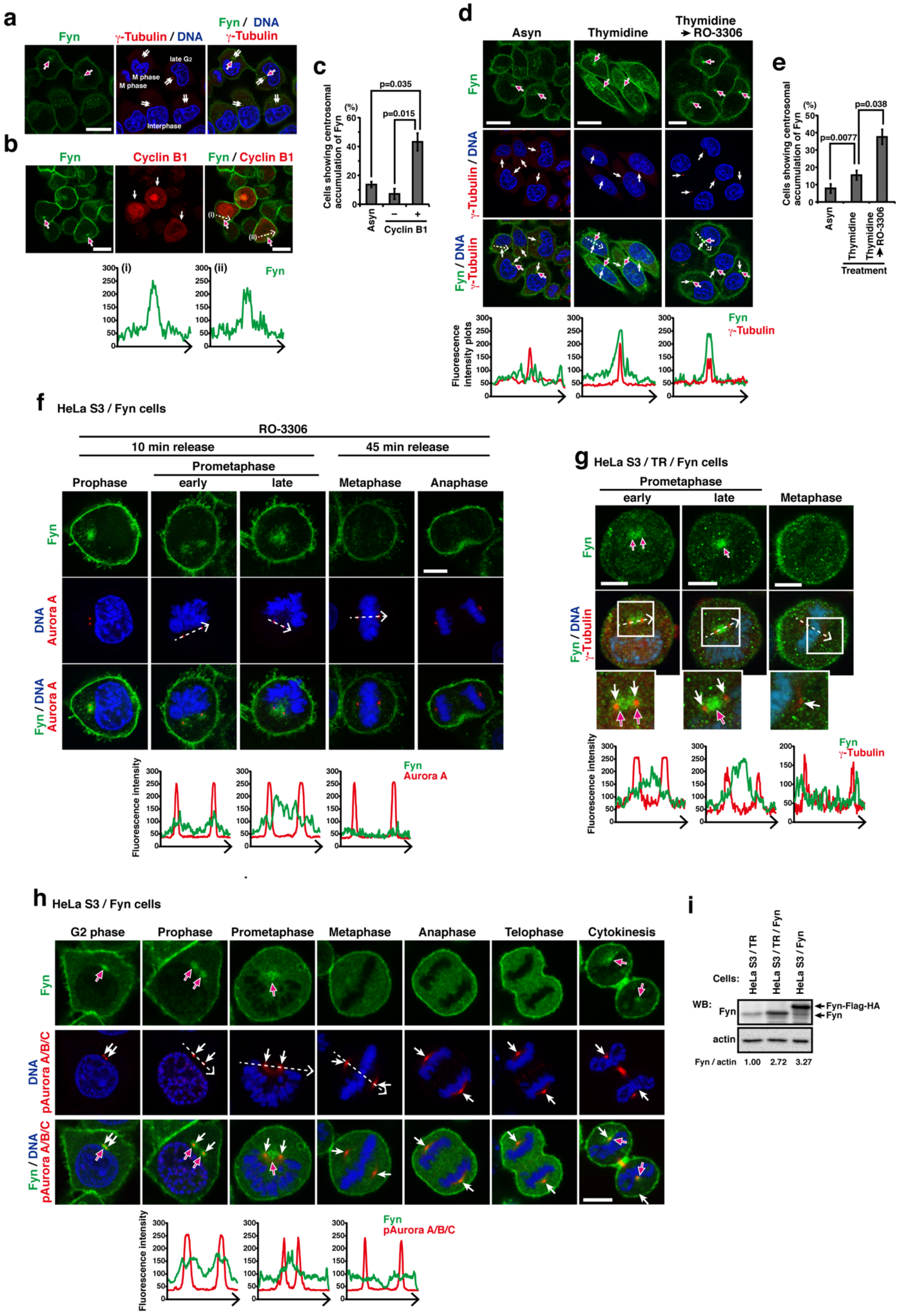

Suppl. Fig. S1. Accumulation of Fyn in the centrosome region during G_2 and M phases.
(a) Asynchronous HeLa S3/Fyn were triply stained for Fyn (green), γ-tubulin (red), and DNA (blue). Cells entering late G_2 and M phases were judged by chromosome condensation. **(b-e) (b,c)** Asynchronous HeLa S3/Fyn cells were doubly stained for Fyn (green) and cyclin B1 (red). Centrosomal Fyn accumulation was quantitated in cyclin B1-positive and -negative cells. Cell counting data was

derived from three independent experiments (n=1052 cells for asynchronous cells, n=687 cells for cyclin B1-negative cells, n=729 cells for cyclin B1-positive cells). Fluorescence intensity plots of Fyn staining along the dotted arrows (i, ii) are shown. **(d,e)** HeLa S3/Fyn cells arrested in G_1/S phase (Thymidine) and late G_2 phase (Thymidine → RO-3306) were triply stained for Fyn (green), γ-tubulin (red), and DNA (blue). Centrosomal Fyn accumulation was quantitated in asynchronous cells and G_1/S- and late G_2-synchronized cells. Cell counting data were derived from three independent experiments (n=918 cells for asynchronous cells, n=1399 cells for thymidine-arrested cells, n=1334 cells for cyclin B1-positive cells). Results (%) represent the means ± S.D. from more than three independent experiments. *p* values were calculated by two-tailed Student's *t*-test. Fluorescence intensity plots of Fyn (green) and γ-tubulin (red) staining along the dotted arrows are shown. **(a-b,d)** Red arrows indicate Fyn accumulation in the centrosome region, and white arrows indicate centrosomes (**a,d**) and cyclin B1-positive cells (**b**). Asyn, asynchronous cells. Scale bars, 20 μm. **(f)** HeLa S3/Fyn cells arrested in late G2 phase by RO-3306 treatment for 20 h were allowed to enter M phase for 15 min or 45 min and triply stained for Fyn, Aurora A (red), and DNA (blue). Fluorescence intensity plots of Fyn and γ-tubulin staining along the dotted lines are shown. Scale bars, 10 μm. **(g)** HeLa S3/TR/Fyn cells arrested in G_1/S phase (double thymidine block) were treated with Dox for inducible expression. Cells were triply stained for Fyn (green), γ-tubulin (red), and DNA (blue). Magnified images of the squared areas are shown in the bottom. Scale bars, 10 μm. **(h)** HeLa S3/Fyn cells were triply stained for Fyn (green), pAurora A/B/C (red), and DNA (blue). Fluorescence intensity plots of Fyn and pAurora A/B/C staining along the dotted arrows are shown in the bottom. Scale bars, 10 μm. **(i)** HeLa S3/TR, HeLa S3/TR/Fyn, and HeLa S3/Fyn cells were treated with or without Dox. Western blotting analysis was performed with the indicated antibodies.

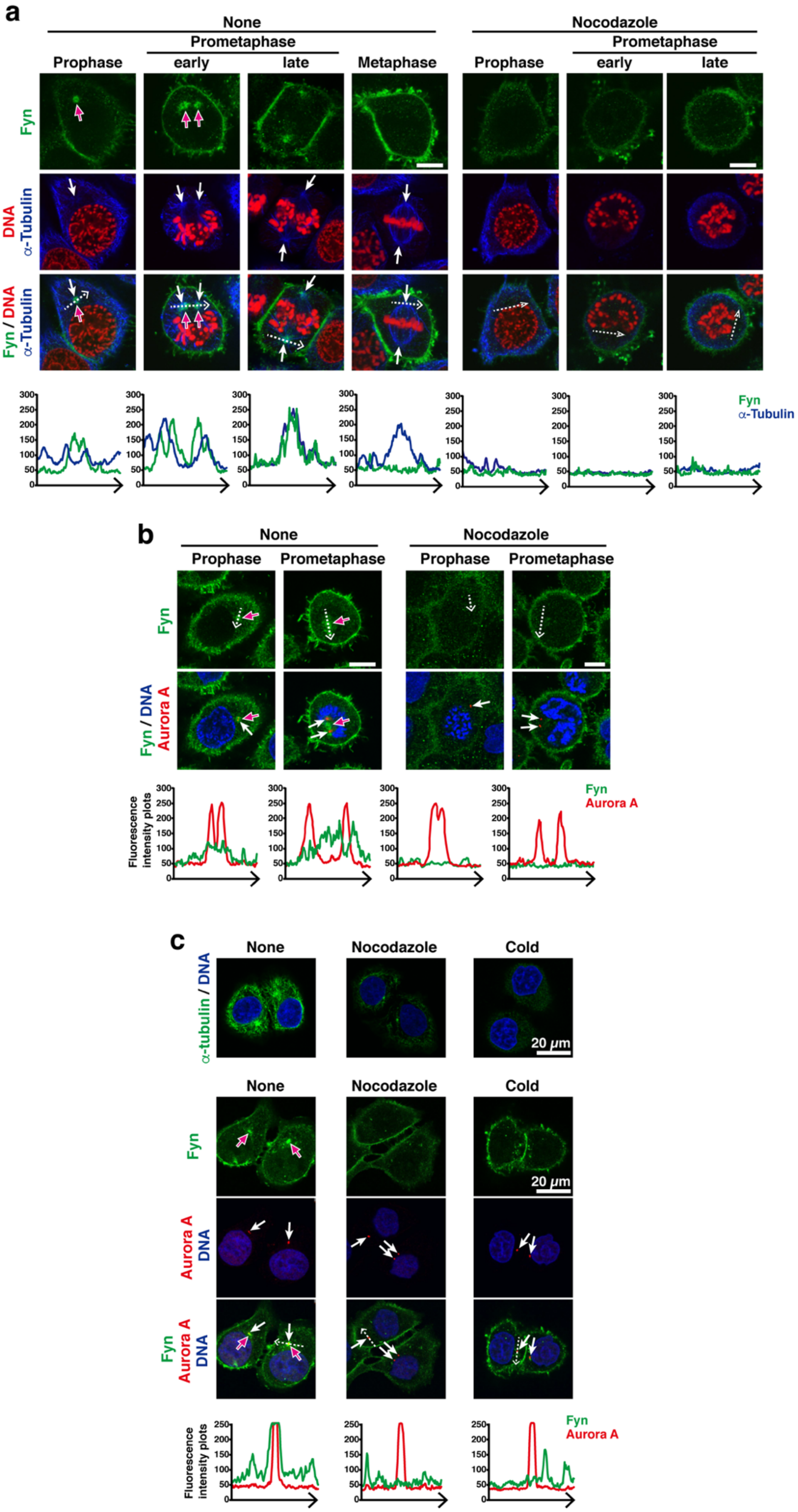

Suppl. Fig. S2. Microtubule-dependent centrosomal accumulation of Fyn.
(a, b) HeLa S3/Fyn cells arrested in late G_2 phase by RO-3306 treatment for 20 h were allowed to enter M phase for 15 min in the presence or absence of 100 ng/ml nocodazole. Scale bars, 10 μm. Cells were triply stained for **(a)** Fyn (green), α-tubulin (blue), and DNA (red) and **(b)** Fyn (green), Aurora A (red),

and DNA (blue). Fluorescence intensity plots of Fyn, α-tubulin, and Aurora A staining along the dotted lines are shown. **(c)** HeLa S3/Fyn cells arrested in late G_2 phase by RO-3306 treatment for 20 h were incubated on ice for the last 2 h (Cold). After cold treatment cells were warmed at 37°C (Cold→37°C). Cells were stained for α-tubulin (green), or doubly stained for Fyn (green) and Aurora A (red) **(a-c)** White and red arrows indicate centrosomes and centrosomal Fyn accumulation, respectively. Scale bars, 10 μm.

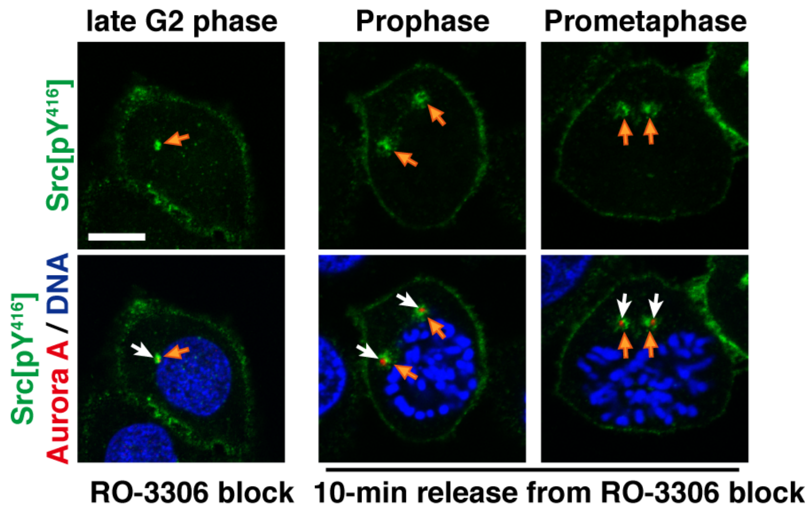

Suppl. Fig. S3. Centrosomal accumulation of kinase-active Src kinases.
HeLa S3/Fyn cells arrested in late G2 phase by RO-3306 treatment for 20 h were allowed to enter M phase for 10 min and triply stained for active Src kinases (Src[pY^{416}], green), Aurora A (red), and DNA (blue). White and orange arrows indicate centrosomes and centrosomal accumulation of active Src kinases, respectively. Scale bar, 10 μm.

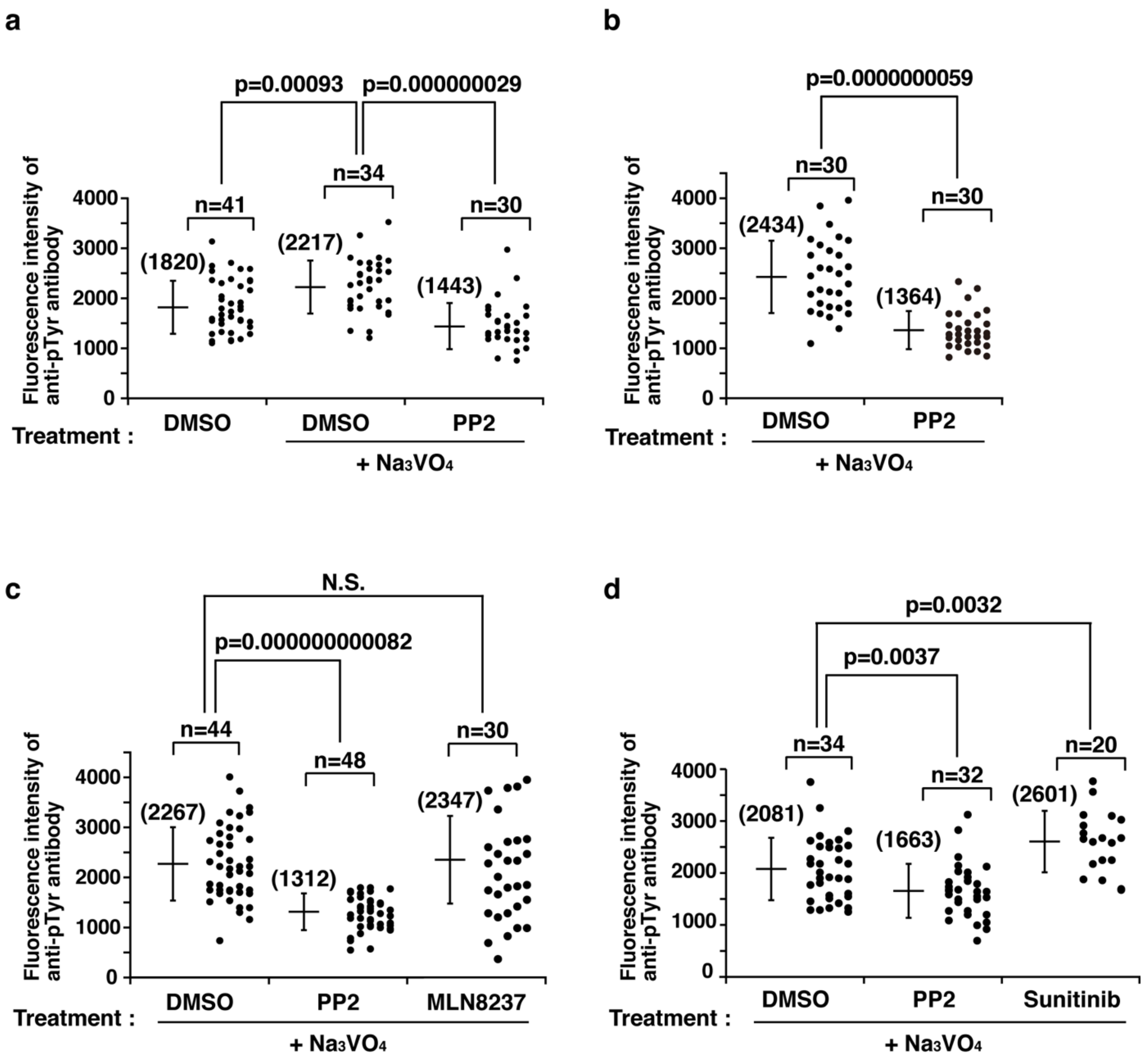

Suppl. Fig. S4. Effect of kinase inhibitors on tyrosine phosphorylation in the centrosome region. **(a)** Parental HeLa S3 cells treated with 20 μM PP2 (Src kinase inhibitor) or 0.1% DMSO (solvent control) for 6 h were fixed/extracted in PTEMF buffer in the presence or absence of 10 mM Na_3VO_4. **(b-d)** Parental HeLa S3 cells were treated **(b)** with 20 μM PP2 or 0.1% DMSO for 12 h, **(c)** with 20 μM PP2, 0.6 μM MLN8237 (Aurora A kinase inhibitor), or 0.1% DMSO for 6 h, or **(d)** with 20 μM PP2, 10 μM sunitinib (multi-targeted receptor tyrosine kinase inhibitor), or 0.1% DMSO for 4 h. Then, cells were fixed/extracted in PTEMF buffer containing 10 mM Na_3VO_4. **(a–d)** The fluorescence intensity of pTyr staining for endogenous proteins in the perinuclear region containing centrosomes was quantitated. The plot represents the integrated fluorescence intensity of pTyr staining acquired from a representative experiment as shown in Fig. 1d. Bars represent means ± S.D. in each experiment, and numbers in parentheses indicate mean values. *p* values were calculated by two-tailed Student's *t*-test. N.S., not significant; n, cell number.

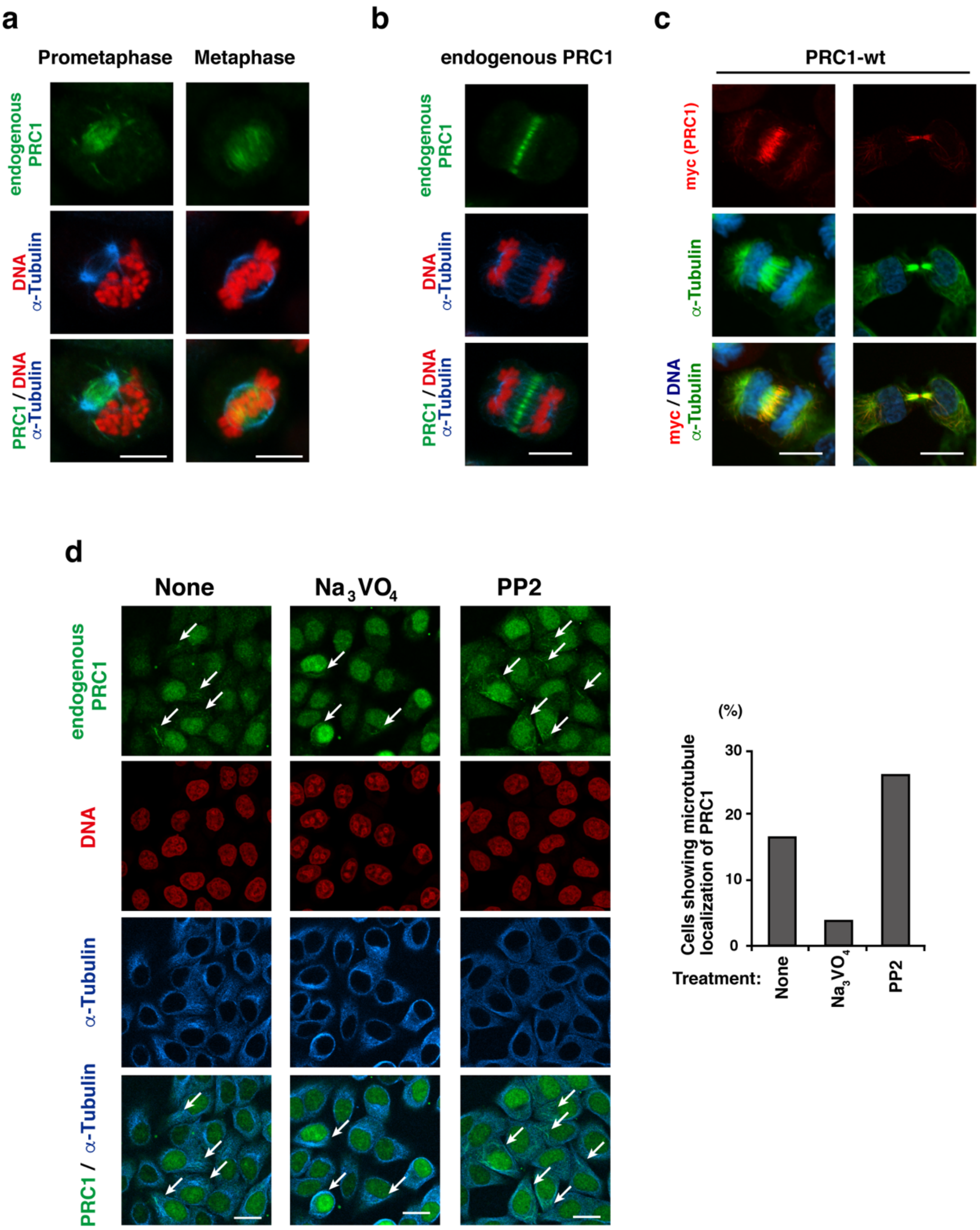

Suppl. Fig. S5. Effect of tyrosine phosphorylation on the microtubule localization of endogenous PRC1.

(**a**) Parental HeLa S3 cells arrested in G_1/S phase (thymidine) and released. Cells in prometaphase and metaphase were stained for PRC1 (green), α-tubulin (red), and DNA (blue). Scale bars, 10 μm. (**b**) Parental HeLa S3 cells arrested in G_1/S phase (thymidine) and released. Cells in late mitosis were stained for PRC1 (green), α-tubulin (red), and DNA (blue). Scale bar, 10 μm. (**c**) HeLa S3/TR cells, which can express myc-PRC1-wt, were treated with Dox for inducible expression. Cells in late mitosis were stained for myc-PRC1 (red), α-tubulin (green), and DNA (blue). Scale bars, 10 μm. (**d**) Parental HeLa S3 cells were treated with 1 mM Na_3VO_4 for 1 h or 10 μM PP2 for 10 h. Cells were stained for PRC1 (green), DNA (red), and α-tubulin (blue). Two independent experiments were performed. The percentage of cells showing the microtubule localization of PRC1 was quantitated (n=151 cells for control, n=202 cells for Na_3VO_4 treatment, n=182 cells for PP2 treatment). Arrows indicate the microtubule localization of PRC1. Scale bars, 20 μm.

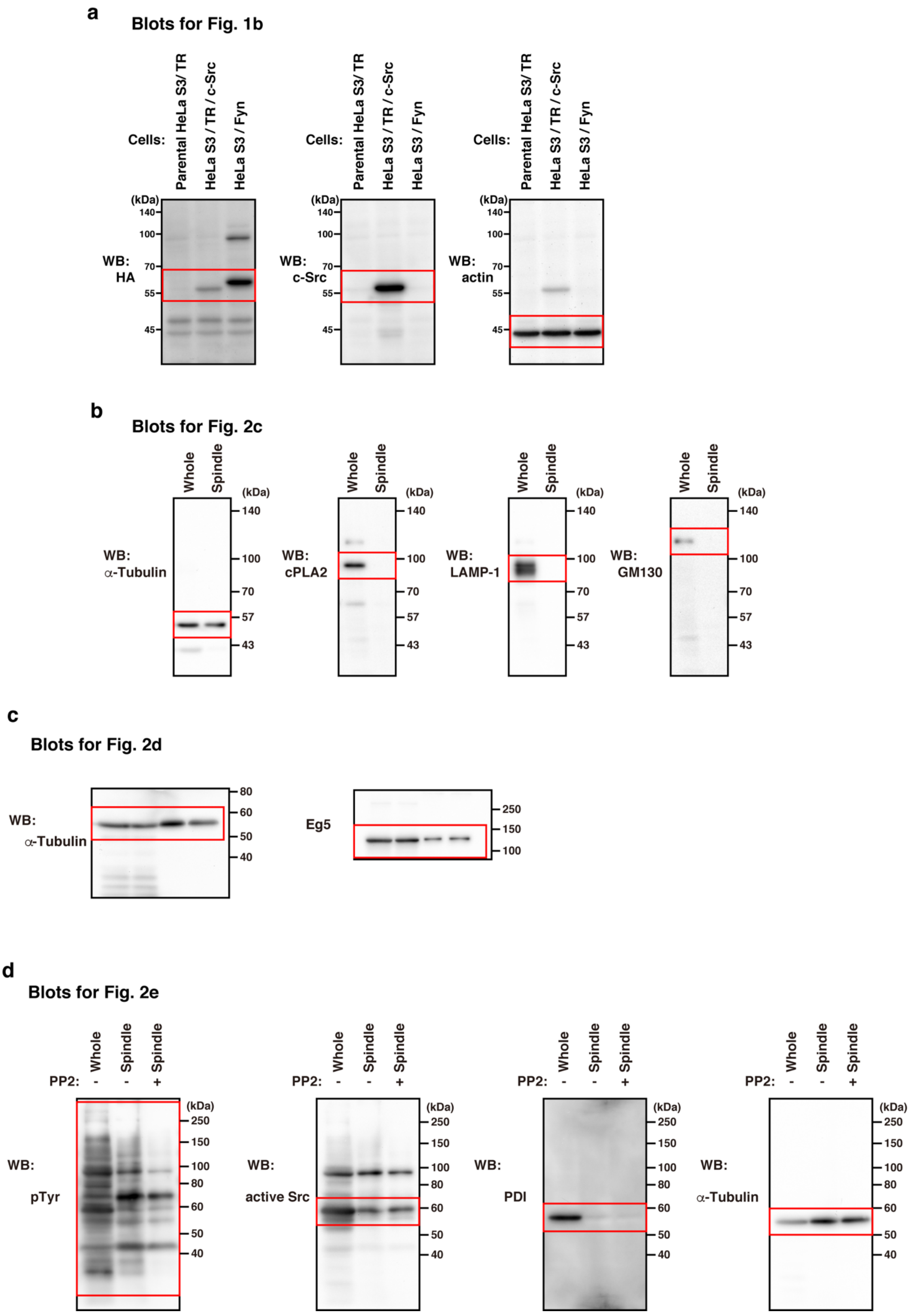

Suppl. Fig. S6. Full-length blots.
Full-length blots for Fig. 1b **(a)**, Fig. 2c **(b)**, Fig. 2d **(c)**, and Fig. 2e **(d)**.

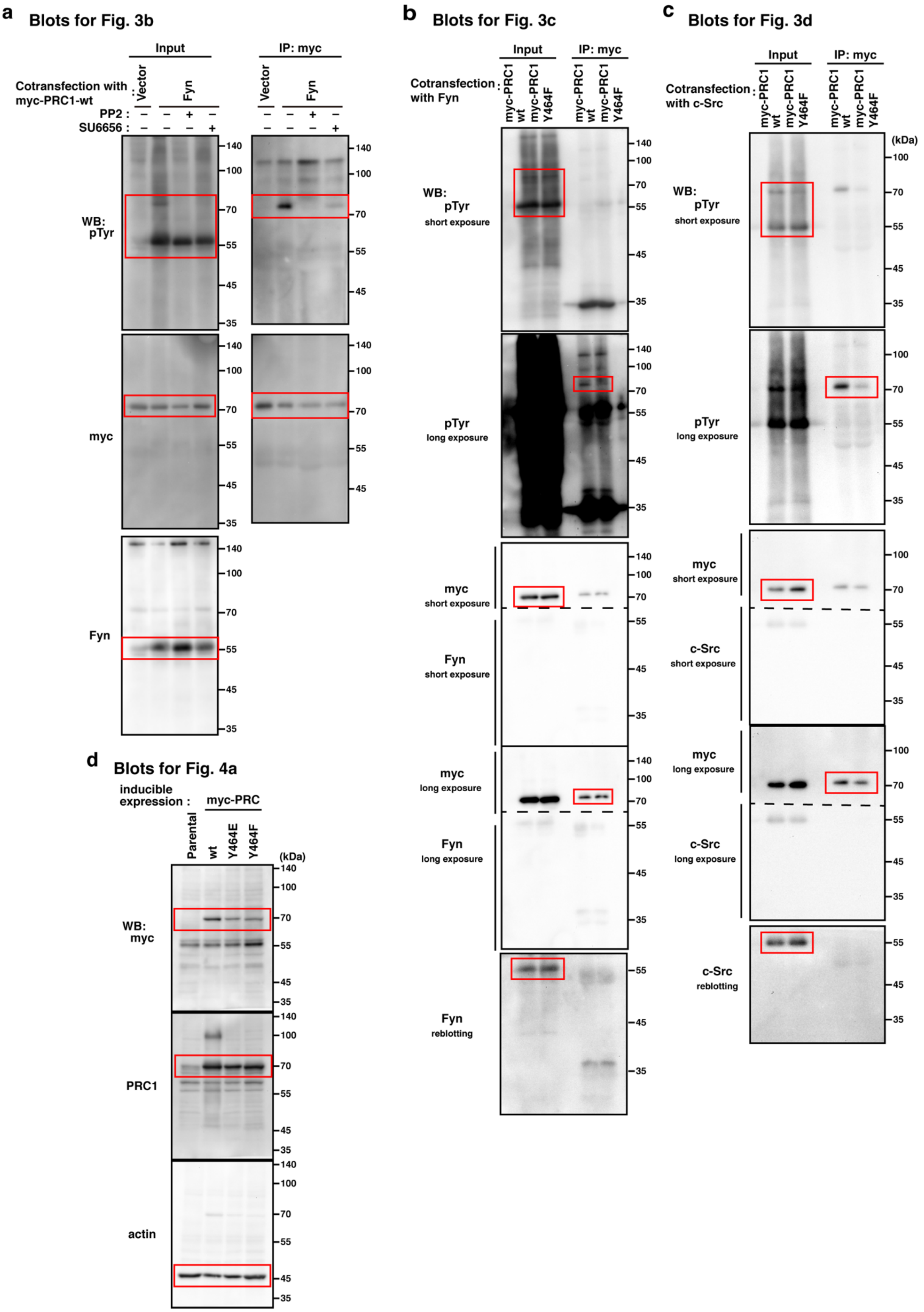

Suppl. Fig. S7. Full-length blots.
Full-length blots for Fig. 3b **(a)**, Fig. 3c **(b),** Fig. 3d **(c)**, and Fig. 4a **(d)**.

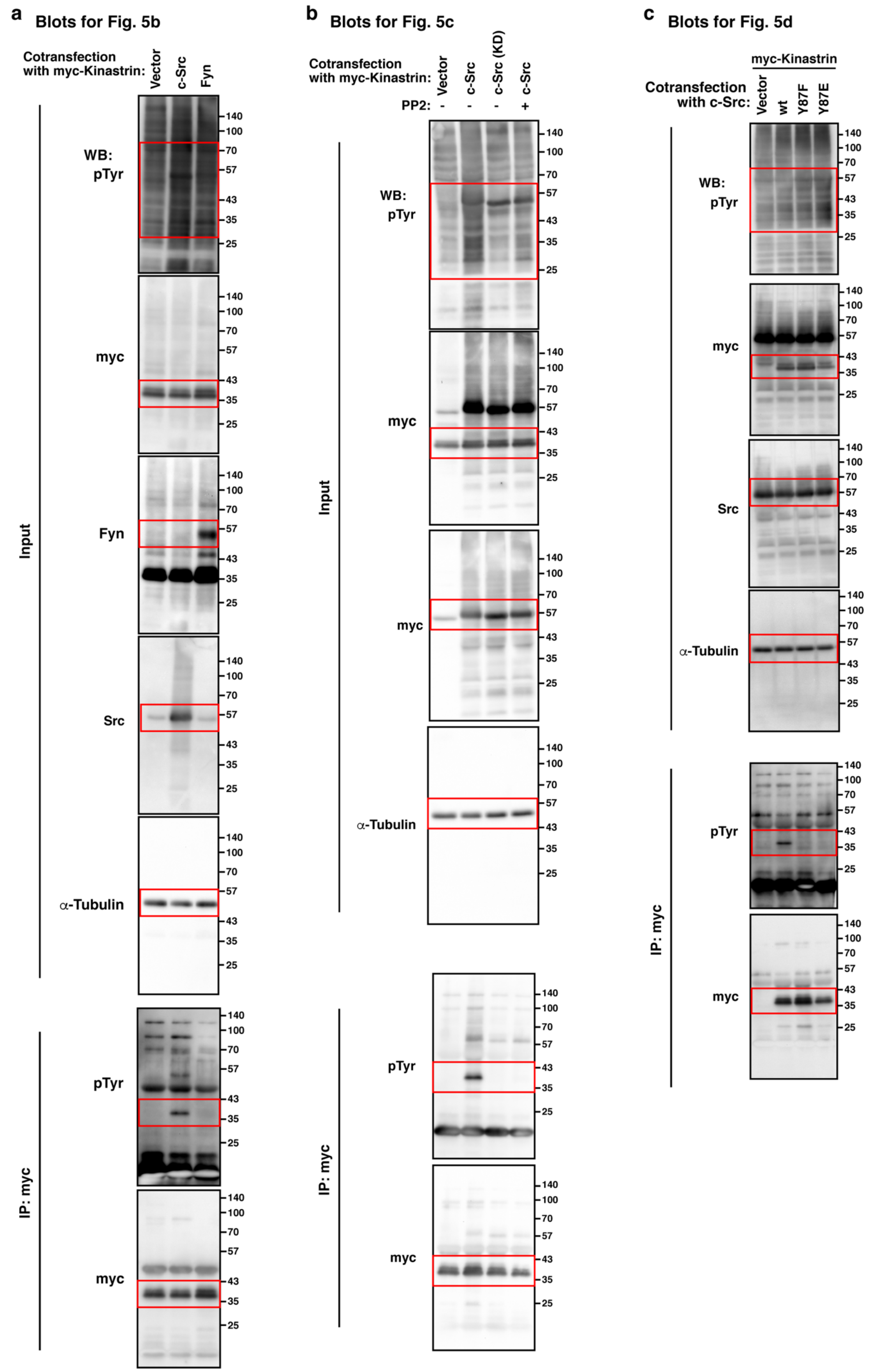

Suppl. Fig. S8. Full-length blots.
Full-length blots for Fig. 5b **(a)**, Fig. 5c **(b)**, and Fig. 5d **(c)**.

Blots for Fig. S1i

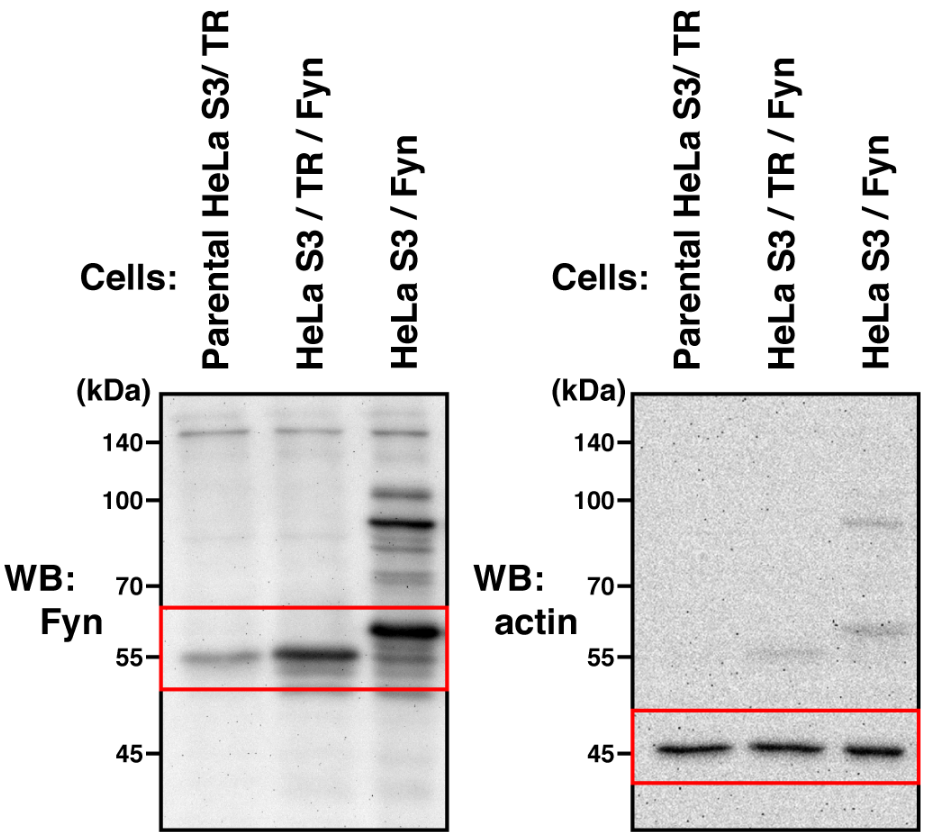

Suppl. Fig. S9. Full-length blots.
Full-length blots for Suppl. Fig. S1i.

Tyrosine-phosphorylated proteins in the isolated mitotic spindle preparation

No.	Phosphorylated peptide	Potential phosphorylation site	Candidate protein name	Accession
1	STSQGSINSPVySR	Y461	Actin-binding LIM protein 1	ABLM1_HUMAN
2	LSLEGDHSTPPSAyGSVK	Y24	Annexin A2	ANXA2_HUMAN
			Putative annexin A2-like protein	ANXA2L_HUMAN
3	AVGASFPLyEPAK	Y340	Antigen KI-67	KI67_HUMAN
4	VRPCVVyGGADIGQQIR	Y301	ATP-dependent RNA helicase DDX3X	DDX3X_HUMAN
5	GVGVALDDPyENYRR	Y894	Calcium homeostasis endoplasmic reticulum protein	CHERP_HUMAN
6	HYEDGYPGGSDNyGSLSR	Y228	Catenin delta-1	CTND1_HUMAN
	APSRQDVyGPQPQVR	Y257		
7	IGEGTyGVVYK	Y15	Cyclin-dependent kinase 1 (CDK1)	CDK1_HUMAN
			Cyclin-dependent kinase 2 (CDK2)	CDK2_HUMAN
			Cyclin-dependent kinase 3 (CDK3)	CDK3_HUMAN
8	IGEGTyGTVFK	Y15	Cyclin-dependent kinase 5 (CDK5)	CDK5_HUMAN
9	FGPyESYDSR	Y74	DBIRD complex subunit ZNF326	ZN326_HUMAN
	SGyGFNEPEQSR	Y93		
10	VyAPASTLVDQPYANEGTVVVTER	Y968	Desmoglein-2	DSG2_HUMAN
11	AESGPDLRyEVTSGGGGTSR	Y28	Desmoplakin	DESP_HUMAN
12	TyQVTPMTPR	Y323	Disks large-associated protein 5	DLGP5_HUMAN
13	VSLEEIySGCTK	Y176	DnaJ homolog subfamily B member 1	DNJB1_HUMAN
		Y172	DnaJ homolog subfamily B member 4	DNJB4_HUMAN
14	GYNDDYyEESYFTTR	Y95	Emerin	EMD_HUMAN
	DSAyQSITHYRPVSASR	Y161		
15	GIyAYGFEKPSAIQQR	Y48	Eukaryotic initiation factor 4A-I	IF4A1_HUMAN
		Y49	Eukaryotic initiation factor 4A-II	IF4A2_HUMAN
		Y54	Eukaryotic initiation factor 4A-III	IF4A3_HUMAN
16	KTEDTyFISSAGKPTPGTQGK	Y1765	Girdin	GRDN_HUMAN
17	TTPSyVAFTDTER	Y43	Heat shock 70 kDa protein 1-like	HS71L_HUMAN
		Y41	Heat shock 70 kDa protein 1A/1B	HS71_HUMAN
		Y42	Heat shock-related 70 kDa protein 2	HS72_HUMAN
		Y41	Heat shock cognate 71 kDa protein	HS7C_HUMAN
		Y43	Heat shock 70 kDa protein 7	HS77_HUMAN
18	AVPKEDIySGGGGGGSR	Y180	Heterogeneous nuclear ribonucleoprotein A0	ROA0_HUMAN
19	SSGPyGGGGQYFAKPR	Y341	Heterogeneous nuclear ribonucleoprotein A1	ROA1_HUMAN
20	SSGSPyGGGYGSGGGSGGYGSR	Y360	Heterogeneous nuclear ribonucleoprotein A3	ROA3_HUMAN
21	DGMDNQGGyGSVGR	Y296	Heterogeneous nuclear ribonucleoprotein H3	HNRH3_HUMAN
22	GSyGDLGGPIITTQVTIPK	Y380	Heterogeneous nuclear ribonucleoprotein K	HNRPK_HUMAN
23	GGNRFEPyANPTKR	Y64	Heterogeneous nuclear ribonucleoprotein M	HNRPM_HUMAN
24	NMGGPYGGGNyGPGGSGGSGGYGGR	Y336	Heterogeneous nuclear ribonucleoproteins A2/B1	ROA2_HUMAN
25	MySYPAR	Y137	Heterogeneous nuclear ribonucleoproteins C1/C2	HNRPC_HUMAN
26	AVCSTyLQSR	Y352	Homeodomain-interacting protein kinase 1	HIPK1_HUMAN
		Y361	Homeodomain-interacting protein kinase 2	HIPK2_HUMAN
27	SLGSVQAPSyGARPVSSAASVYAGAGGSGSR	Y24	Keratin, type I cytoskeletal 18	K1C18_HUMAN
	SLGSVQAPSYGARPVSSAASVyAGAGGSGSR	Y36		
28	LSSARPGGLGSSSLyGLGASRPR	Y40	Keratin, type II cytoskeletal 7	K2C7_HUMAN
	SAyGGPVGAGIR	Y55		
29	KAQSNPyYNGPHLNLK	Y185	LIM domain only protein 7	LMO7_HUMAN
	WIDATSGIyNSEK	Y1142		
30	KLDVEEPDSANSSFySTR	Y1836	Nuclear mitotic apparatus protein 1 (NuMA1)	NUMA1_HUMAN
31	ERDyAEIQDFHR	Y1080	Partitioning defective 3 homolog	PARD3_HUMAN
	EGHMMDALyAQVK	Y1127		
	TySFEQPWPNARPATQSGR	Y1177		
32	SFySSHYAR	Y112	Periphilin-1	PPHLN_HUMAN
33	GGVGSRADyDTLSLR	Y176	Plakophilin-3	PKP3_HUMAN
34	QQGLASyDYVR	Y3777	Plectin	PLEC_HUMAN
	GYySPYSVSGSGSTAGSR	Y4612		
	GYYSPySVSGSGSTAGSR	Y4615		
35	VVQNDAyTAPALPSSIR	Y236	Polymerase delta-interacting protein 3	PDIP3_HUMAN
36	SFTPDHVVyAR	Y308	Polymerase I and transcript release factor	PTRF_HUMAN
37	TGAPQyGSYGTAPVNLNIK	Y110	Pre-mRNA 3'-end-processing factor FIP1	FIP1_HUMAN
	TGAPQYGSyGTAPVNLNIK	Y113		
38	TGTAyTFFTPNNIK	Y442	Probable ATP-dependent RNA helicase DDX5	DDX5_HUMAN
39	AKEISEASENIySDVR	Y1790	Protein ELYS	ELYS_HUMAN
40	LSSATANALySSNLRDDTK	Y1032	Protein FAM83H	FA83H_HUMAN
41	AQIPEGDyLSYR	Y1104	Protein LAP2	LAP2_HUMAN
42	QTETEMLyGSAPR	Y464	Protein regulator of cytokinesis 1 (PRC1)	PRC1_HUMAN
43	KKEADSVyGEWVPVEK	Y2192	Protein SON	SON_HUMAN
44	TAATSVPAyEPLDSLDR	Y608	Putative RNA-binding protein 15	RBM15_HUMAN
45	NEEENIySVPHDSTQGK	Y1105	Rho GTPase-activating protein 35	RHG35_HUMAN
46	GGHMDDGGySMNFNMSSSR	Y134	RNA-binding motif protein, X chromosome	RBMX_HUMAN
	SDLySSGRDR	Y335		
47	ASyVAPLTAQPATYR	Y226	RNA-binding protein 14	RBM14_HUMAN
	TQSSASLAASyAAQQHPQAAASYR	Y528		
	LAELSDyRR	Y614		
	LPDAHSDyAR	Y645		
	ySGSYNDYLR	Y648		
	YSGSYNDyLR	Y655		
48	VADLTEQYNEQyGAVR	Y194	RNA-binding protein 4	RBM4_HUMAN
49	NLVPGESVyGEKR	Y118	rRNA 2'-O-methyltransferase fibrillarin	FBRL_HUMAN
50	FEDPRDAEDAIyGR	Y70	Serine/arginine-rich splicing factor 9	SRSF9_HUMAN
51	SGDSEVyQLGDVSQK	Y73	Single-stranded DNA-binding protein, mitochondrial	SSBP_HUMAN
52	TVySLQPPSALSGGQPADTQTR	Y87	Small kinetochore-associated protein (kinastrin / SKAP)	SKAP_HUMAN
53	AADKLAPAQyIR	Y176	SNW domain-containing protein 1	SNW1_HUMAN
54	AQPVPHyGVPFKPQIPEAR	Y519	Targeting protein for Xklp2	TPX2_HUMAN
55	TFLRPSPEDEAIyGPNTK	Y506	Tight junction protein ZO-2	ZO2_HUMAN
56	INVyYNEATGGK	Y50	Tubulin beta-4B chain	TBB4B_HUMAN
57	LIEDNEyTAR	Y420	Tyrosine-protein kinase Fyn	FYN_HUMAN
	WTAPEAALyGR	Y440		
58	TySLGSALRPSTSR	Y38	Vimentin	VIME_HUMAN
	SLyASSPGGVYATR	Y53		
	SLYASSPGGVyATR	Y61		

Classification of the tyrosine-phosphorylated proteins identified in the isolated mitotic spindle preparation

Classification	
spindle / microtubule interactor & tubulin	6 (10.3%)
actin / intermediate filament (IF) interactor & IF	8 (13.8%)
membrane interactor	11 (19.0%)
RNA interactor	23 (39.7%)
DNA / chromosome interactor	4 (6.9%)
protein kinase (including Fyn)	4 (6.9%)
chaperone	2 (3.4%)
Total	58 (100%)

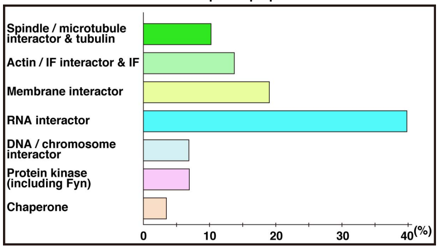

Suppl. Table S1. Identification of tyrosine-phosphorylated proteins in the isolated mitotic spindle preparation.

The trypsinized peptides derived from tyrosine-phosphorylated proteins associated with mitotic spindles were identified by LC/MS/MS (see Methods). A number of tyrosine-phosphorylated proteins associated with mitotic spindles were identified, and tyrosine-phosphorylated peptide fragments actually obtained were also shown in the right columns. We classified the isolated tyrosine-phosphorylated proteins as several groups (bottom panels). Note that Fyn was detected in the mitotic spindle preparation.

	Forward primer (5' → 3')	Reverse primer (5' → 3')
PRC1 cDNA	GGTGCTTGTACAATGAGGAGAAGTGAGGTGCTGGCGGAG	CGCACCCTCGAGTCAGGACTGGATGTTGGTTGAATTGAGGATTCCAG
PRC1 Y464E	CAAAAAACAGACAGAGACAGAGATGCTCGAGGGCAGCGCTCCTCGAAC	TTGCTAGGTGTTCGAGGAGCGCTGCCCTCGAGCATCTCTGTCTC
PRC1 Y464F	GACAGAGACAGAGATGCTGTTTGGCAGCGCTCCGCGGACACCTAG	CGCTTGCTAGGTGTCCGCGGAGCGCTGCCAAACAGCATCTCTG
Kinastrin cDNA	AGCTACGGTACCTCCAGTATGGCGGCTCCCGAAGC	AGCGATCTCGAGGCCACTTGCTTCTTCTTACATTTCTAATAGCTGCTC
Kinastrin Y87E	CAGTGTGGTTAAGACAGTGGAAAGCTTGCAGCCCCCCTCTGCGCTGAGC	CAGAGGGGGGCTGCAAGCTTTCCACTGTCTTAACCACACTGGTCATCG
Kinastrin Y87F	CAGTGTGGTTAAGACAGTGTTTAGTCTACAGCCCCCCTCTGCGCTGAG	CAGAGGGGGGCTGTAGACTAAACACTGTCTTAACCACACTGGTCATCG

Suppl. Table S2. Primer list for PRC1 and kinastrin.
